# *Polycomb* Controls Gliogenesis by Regulating the Transient Expression of the Gcm/Glide Fate Determinant

**DOI:** 10.1371/journal.pgen.1003159

**Published:** 2012-12-27

**Authors:** Anna Popkova, Roberto Bernardoni, Celine Diebold, Véronique Van de Bor, Bernd Schuettengruber, Inma González, Ana Busturia, Giacomo Cavalli, Angela Giangrande

**Affiliations:** 1 Institut de Génétique et de Biologie Moléculaire et Cellulaire, CNRS/INSERM/UDS, Illkirch, France; 2 Dipartimento di Farmacia e Biotecnologie (FaBit), Health Sciences and Technologies–Interdepartmental Center for Industrial Research (HST-ICIR), Università di Bologna, Bologna, Italy; 3 Institut de Génétique Humaine, CNRS, Montpellier, France; 4 Centro de Biologia Molecular, CSIC–UAM, Madrid, Spain; University of British Columbia, Canada

## Abstract

The Gcm/Glide transcription factor is transiently expressed and required in the *Drosophila* nervous system. Threshold Gcm/Glide levels control the glial versus neuronal fate choice, and its perdurance triggers excessive gliogenesis, showing that its tight and dynamic regulation ensures the proper balance between neurons and glia. Here, we present a genetic screen for potential *gcm/glide* interactors and identify genes encoding chromatin factors of the Trithorax and of the Polycomb groups. These proteins maintain the heritable epigenetic state, among others, of HOX genes throughout development, but their regulatory role on transiently expressed genes remains elusive. Here we show that Polycomb negatively affects Gcm/Glide autoregulation, a positive feedback loop that allows timely accumulation of Gcm/Glide threshold levels. Such temporal fine-tuning of gene expression tightly controls gliogenesis. This work performed at the levels of individual cells reveals an undescribed mode of Polycomb action in the modulation of transiently expressed fate determinants and hence in the acquisition of specific cell identity in the nervous system.

## Author Summary

Epigenetic mechanisms are essential to define cell identity, and the Polycomb and the Trithorax Group proteins (PcG and TrxG, respectively) control the body plan by maintaining the epigenetic state of homeotic genes. PcG and TrxG act by triggering stable chromatin modifications that are “remembered” after cell division and keep gene expression in an OFF or ON state. Recent genome-wide analyses call for additional targets of PcG proteins, but the role of these chromatin factors in dynamic transcriptional states and/or in specific cell fates is difficult to apprehend, mostly because very sensitive readouts are required. This in vivo study performed at the single-cell level shows that PcG proteins affect the levels and the kinetics of the transiently expressed *Drosophila* glial determinant and transcription factor Gcm/Glide. Thus, PcG proteins also finely tune gene expression, and this is independent of memory mechanisms, suggesting that “transient” promoters may have a different affinity to PcG proteins compared to “stable” promoters. PcG proteins negatively affect Gcm/Glide autoregulation, thereby promoting neurogenesis at the expense of gliogenesis. Thus, PcG genes act in the fate choice between two types of differentiated cells, implying that distinct cell populations have specific requirements for general chromatin modifiers.

## Introduction

One of the most challenging issues in biology is to elucidate the mechanisms underlying cell fate determination and maintenance. The *Drosophila melanogaster* Glial cell missing/Glial cell deficient transcription factor (Gcm/Glide, referred throughout the text to as Gcm) is transiently expressed and is key to decide between glial and neuronal fates in the multipotent neural precursors [1]–[6]. Threshold levels of Gcm are necessary and sufficient to induce gliogenesis and the tight regulation of its expression prevents defective/excessive gliogenesis [7]–[11]. These features make Gcm an ideal tool to study cell differentiation and plasticity.

Two major classes of proteins that modify the chromatin structure and its condensation state, the Polycomb group (PcG) and the Trithorax group (TrxG), are known as critical regulators of HOX transcription factors, which act as molecular switches that are maintained in a silent or in an active state [12]. PcG and TrxG proteins act in large multimeric complexes that bind specific DNA regions called Polycomb (or Trithorax) response elements (respectively PREs and TREs) [13]. PcG and TrxG complexes trigger posttranslational modification of histone tails that have opposite effects on gene activity, mainly methylation of H3K27 induced by PcG complexes (negative regulation) and methylation of H3K4, H3K36 as well as acetylation of H3K27 by TrxG complexes (positive regulation) ([12], [14] and references therein). PcG proteins enter two main conserved complexes called Polycomb Repressive Complex 1 and 2 (PRC1 and PRC2). The latter is formed by four core components including, in flies, Enhancer of zeste (E(z)), and catalyzes the reaction that leads to di- and tri-methylation of H3K27. This epigenetic mark is recognized by Polycomb (Pc), which belongs to the PRC1 complex.

Recent chromatin immunoprecipitation studies have shown that PcG and TrxG binding is also associated with dynamic transcriptional states modulating different processes including mitogenic pathways and progression from multipotency to differentiation ([12], [15]–[19] and references therein). Understanding the mode of action of PcG and TrxG proteins in dynamic processes, however, requires analyses at the level of identified cells and times. This is particularly important for developmental genes that are expressed transiently and in specific cell populations. The present in vivo study analyzes the role of Pc in fly gliogenesis.

To identify components and regulators of the Gcm pathway, we designed a screen for genetic modifiers of a dominant phenotype due to *gcm* ectopic expression and identified PcG and TrxG proteins. Importantly, mutations in PcG components and in TrxG members found in chromatin remodeling complexes enhance the *gcm* dominant phenotype, whereas mutations in TrxG proteins known to specifically counteract PcG function rescue it. This suggests that a balanced action of these chromatin modifiers regulate Gcm function. Moreover, we demonstrate that the *gcm* regulatory sequences carry a PRE and are bound by Pc. Finally, Pc inhibits the autoregulatory loop ensuring threshold Gcm levels [7] and hence gliogenesis.

To our knowledge, this is the first direct evidence that PcG proteins negatively modulate a transiently expressed fate determinant, thereby affecting a specific lineage in the nervous system.

## Results

### A screen to identify *gcm* genetic interactors

**Figure 1:**
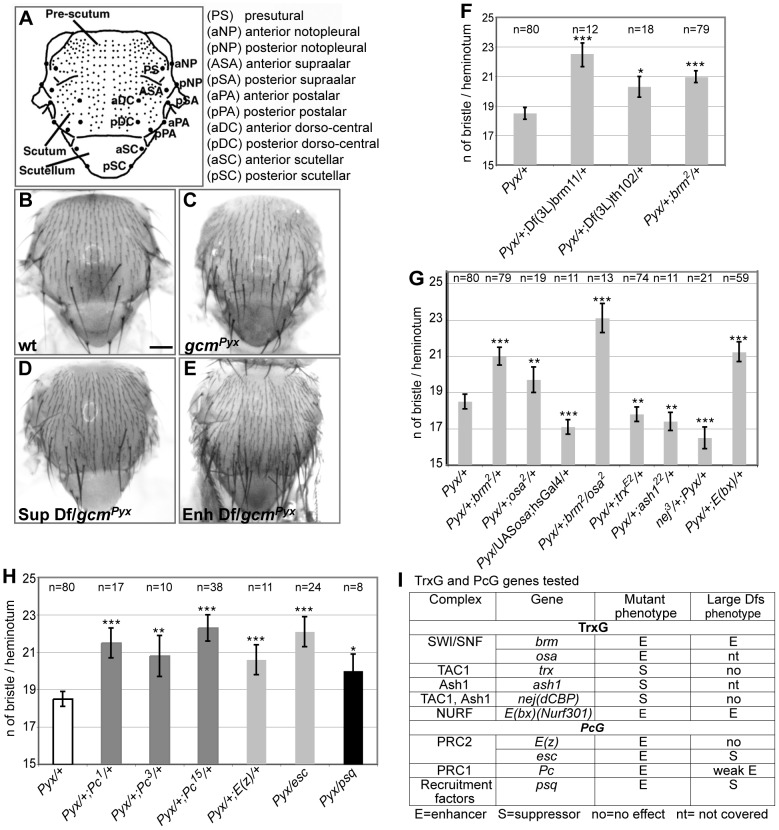
Genetic screen for *gcm^Pyx^* modifiers and interactions with TrxG and PcG proteins. (A) Drawing of an adult notum. Small and large dots represent microchaetae and macrochaetae, respectively. Macrochaete symbols to the right. (B–E) Adult nota from wt (WT; B), *gcm^Pyx^/+* (C), *gcm^Pyx^*/suppressor deficiency (D), *gcm^Pyx^*/enhancer deficiency (E) flies. Dfs = Deficiencies. Scale bar = 200 µm. Histograms present the average number of bristles per heminotum (y-axis) in different genotypes (x-axis). In all figures, average values are indicated +/− SEM (bars); *P-*values from t-test are indicated in the following way: *** (*P*≤10^−3^), ** (*P*≤10^−2^), * (P≤5×10^−2^). *Pyx* stands for *gcm^Pyx^*. (F) Deficiencies deleting *brm*, (*Df(3L)brm11* and *Df(3L)th102*), as well as the *brm* mutation. *P-*values vs. *gcm^Pyx^*/+: *gcm^Pyx^*/+; *Df(3L)brm11/+* (8,9×10^−6^); *gcm^Pyx^/+*; *Df(3L)th102/+* (0,02); *gcm^Pyx^*/+; *brm/+* (4,2×10^−7^). (G) *gcm^Pyx^* interaction with *trxG* genes. *P-*values vs. *gcm^Pyx^*/+: *gcm^Pyx^*/+; *brm/+* (4,2×10^−7^); *gcm^Pyx^*/+; *osa/+* (0,002); *gcm^Pyx^*/*UAS-osa; hsGal4/+* (0,0007); *gcm^Pyx^*/+; *brm*/*osa* (3,4×10^−8^); *gcm^Pyx^*/+; *trx/+* (0,009); *gcm^Pyx^/+*; *ash1*/+ (0,01); *nej*/+; *gcm^Pyx^/+* (6,9×10^−7^); *gcm^Pyx^*/+; *E(bx)/+* (1,3×10^−5^). (H) *gcm^Pyx^* interaction with *PcG* genes. Color code indicates members of the same complex (dark gray: PRC1, pale gray: PRC2, black: PRC recruiter). *P-*values vs. *gcm^Pyx^*/+: *gcm^Pyx^*/+; *Pc^1^*/+ (6,2×10^−6^); *gcm^Pyx^*/+; *Pc^3^*/+ (0,0022); *gcm^Pyx^*/+; *Pc^15^*/+ (1,5×10^−11^); *gcm^Pyx^*/+; *E(z)*/+ (0,001); *gcm^Pyx^*/*esc* (2,6×10^−8^); *gcm^Pyx^*/*psq* (0,03). (I) Summary of the tested TrxG and PcG mutations. From left to right: the biochemical complex, the genes within the complex, the mutant phenotype over *gcm^Pyx^* (No – no effect; S – suppressor; E – enhancer) and the large deficiency phenotype (nt – gene region not covered by the kit).

The need of tight Gcm regulation prompted us to screen for interactors using a sensitized background. This approach allows the dissection of molecular cascades when the loss of a gene product is embryonic lethal. The *Drosophila* thorax (notum) carries a stereotyped number of sensory organs called macrochaete or bristles. *gcm^Pyx^*/+ flies ectopically express *gcm* in the larval notum, which triggers the differentiation of supernumerary sensory organ precursors (SOPs) and bristles (Figure 1A–1C) [20]. *gcm^Pyx^*/+ females show, in average, 18,5 bristles instead of the 11/heminotum typical of wild-type (wt) animals. Using large overlapping deficiencies (67–75% genome coverage, Deficiency kit, Bloomington), we performed a primary screen and identified 42 genomic regions that dominantly enhance or suppress the *gcm^Pyx^* dominant phenotype when deleted (Figure 1D–1E, Figure S1Aa and S1B). These regions were selected for quantitative analyses (Figure S1Ab), which identified weakly and strongly modifying deficiencies. We further analyzed the latter ones (Figure S1Ab, S1B) and identified 28 interacting genomic regions. A secondary quantitative screen with smaller deficiencies (Figure S1Ac, Table 1) allowed us to identify those that act as strong modifiers, based on statistical analyses. Single gene loss of function mutations within those deficiencies were then analyzed and the interaction was confirmed for 18 of them (Figure S1Ad, Figure S2, Table 1). In sum, the Deficiency kit allowed us to identify large interacting regions and to progressively refine the analysis to single mutations.

**Table 1:** Small deficiencies tested in the secondary screen.

Large Df name	Small Df name	Cytology	Phenotype over *gcm^Pyx^*	Tested candidate gene name and phenotype over *gcm^Pyx^*
**Chromosome arm X**				
Df(1)JF5	Df(1)ED418	5C7;5E4	No	
Df(1)RK4	Df(1)ED7265	12F4;13A5	No	
**Chromosome arm 2L**				
Df(2L)C144	Df(2L)N6	23A6;23B1	Weak S	
	Df(2L)JS17	23C1-C2;23E1-E2	S	**lilli**(S)
Df(2L)cl-h3, Df(2L)E110	Df(2L)cl7	25D7;26A7	S	**mid**(S)
Df(2L)E110	Df(2L)Exel6016	26C1;26D1	No	
Df(2L)J39	Df(2L)J2	31B1;32A1-32A2	S	**pim**(S)
	Df(2L)J3	31D;31F2-F5	No	**pim**(S)
	Df(2L)J1	31B1;31D8-D11	No	**pim**(S)
	Df(2L)ED746	31F4;32A5	No	
	Df(2L)Exel8026	31F5;32B3	No	**UbcD2**(No)
	Df(2L)Exel7049	32B1;32C1	No	
	Df(2L)Exel6027	32D2;32D5	Lethal	**l(2)gd1**(S)
Df(2L)Prl	Df(2L)esc-P2-0	33A1;33B1-33B2	S	**crol**(S)
Df(2L)TE35BC-24	Df(2L)el80f1	34E3;35D2-D5	S	
	Df(2L)r10	35D1;36A6-A7	S	**esg**(S), **wor**(No), **sna**(S)
	Df(2L)ED3	35B2;35D1	S	**Su(H)** (S), **esg** * (S)
	Df(2L)ED1050	35B8;35D4	S	**esg**(S), **wor**(No), **sna**(S)
	Df(2L)Exel8034	35C5;35D2	No	**esg**(S), **wor**(No)
	Df(2L)Exel7063	35D2;35D4	S	**wor**(No), **sna**(S)
Df(2L)TW50	Df(2L)E71	36F2-F6;37C6-D1	S	**brat**(S)
	Df(2L)Sd77	37C6-D1;38C1-C2	Weak S	
	Df(2L)ED1231	37C5;37E3	No	
	Df(2L)ED1303	37E5;38C6	S	
Df(2L)TW84	Df(2L)ED1305	38B4;38C6	No	
	Df(2L)ED1315	38B4;38F5	S	
	Df(2L)Exel6046	38C2;38C7	S	
	Df(2L)DS6	38E2;39E7	S	**E2f2**(S)
**Chromosome arm 2R**				
Df(2R)M41A4	Df(2R)rl10a	h38R-h41;h41-41A3	Weak E	
Df(2R)E3363	Df(2R)ED2076	47A10-47C1	S	**lola**(S)
	Df(2R)Exel6059	47C5;47D6	S	
Df(2R)CX1	Df(2R)Exel6062	49E6;49F1	No	
	Df(2R)Exel7128	50C5;50C9	S	**Ago1**(S)
**Chromosome arm 3L**				
Df(3L)emc-E12	Df(3L)ED4079	61A5;61B1	Weak E	
	Df(3L)Exel6083	61A6;61B2	S	
	Df(3L)Exel6084	61B2-61C1	No	**E(bx)**(E)
	Df(3L)ED4177	61C1;61E2	E	
	Df(3L)Exel6085	61C3;61C9	S	
Df(3L)pbl-X1	Df(3L)RM5-2	65E;66A17	E	
Df(3L)fz-M21	Df(3L)ED4543	70C6;70F4	No	
	Df(3L)Exel6122	70D4;70D7	S	
	Df(3L)Exel6123	70D7;70E4	No	
	Df(3L)Brd12	70E;71A2	weak S	
	Df(3L)ED217	70F4;71E1	S	
	Df(3L)Brd15	71A1-A2;71C1-C2	weak S	
	Df(3L)Exel6125	71A3;71B3, 5	S	
Df(3L)brm11	Df(3L)th102	72A2;72D10	E	**brahma** (E)
**Chromosome arm 3R**				
Df(3R)2-2	Df(3R)ED5021	81F6;82A5	No	**hkb**(weakS)
	Df(3R)XM3	82A3-A6;82B	S	**hkb**(weakS)
	Df(3R)Z1	82A5-A6;82E4	weakE	
	Df(3R)ED5066	82C5;82E4	E	
Df(3R)Cha7	Df(3R)DG2	89E-F;91B1-B2	E	**repo**(weakE)
	Df(3R)Exel6178	90F4;91A5	E	**repo**(weakE)
	Df(3R)ED2	91A5;91F1	E	**fru**(weakE)
	Df(3R)Exel6179	91A5;91B5	E	**fru**(weakE)
Df(3R)3450	Df(3R)Exel6210	98E1;98F5	No	
	Df(3R)Exel6211	98F5;98F6	No	
Df(3R)awd-KRB	Df(3R)E40	100C5;100F1-F5	E	**ttk** (E)

* only upstream region.

From the primary screen to the genes. From left to right, columns indicate the name of the large deficiencies identified in the primary quantitative screen, the name of the small deficiencies in that region, their cytology, the phenotype observed over *gcm^Pyx^* (No – no effect; S – suppressor; E – enhancer), the name and phenotype of putative interactors genes analyzed over *gcm^Pyx^*.

To evaluate the specificity and the sensitivity of the screen, we asked whether the selected deficiencies eliminate genes expected to interact with *gcm*. The *gcm^Pyx^* phenotype correlates with the ectopic formation of proneural territories and precursors of the central (CNS) and peripheral (PNS) nervous systems, neuroblasts (NB) and SOPs, respectively [21], [22]. Thus, mutations of NB/SOP specific genes should act as *gcm^Pyx^* suppressors and indeed, the large and the small deficiencies covering three genes *– escargot (esg)*, *worniu (wor)* and *snail (sna) –* expressed in most embryonic NBs act as *gcm^Pyx^* suppressors (Table 1, Figure S1C). Testing single gene loss of functions confirmed that *sna* and *esg* mutations act as *gcm^Pyx^* suppressors. Accordingly, *esg* overexpression triggers the opposite phenotype (Figure S1C). Finally, genes as *pimples* (*pim*) and *crooked legs* (*crol*), identified in a microarray as induced by Gcm [23], were also identified in our screen (Figure S1D).

The fact that known and predicted *gcm* interactors were identified validates our screen and shows that the dominant bristle phenotype is a reliable and very sensitive readout.

### The *Pc* and the *trx* group mutations interact with *gcm^Pyx^*

A genomic region identified in the screen covers the *trxG* gene *brahma* (*brm*), which encodes a transcriptional coactivator related to yeast SWI/SNF proteins and plays a role in ATP-dependent nucleosomal remodeling [24]. The large and the small deficiencies covering *brm*, *Df(3L)brm11*, *Df(3L)th102* and, most importantly, a null *brm* allele, enhance the *gcm^Pyx^* phenotype (Figure 1F). To extend our findings, we tested *osa*, an integral component of the Brahma complex [25]. *osa* loss of function also enhances the *gcm^Pyx^* phenotype, whereas *osa* gain of function (GOF: *hs-Gal4;UAS-osa* flies) suppresses it (Figure 1G, 1I). Thus, *osa* acts as *brm*, moreover, double *brm/osa* heterozygous mutants show an even stronger phenotype.

Furthermore, a deficiency covering *Enhancer of bithorax (E(bx))* and the *E(bx)* mutation itself enhance the *gcm^Pyx^* phenotype (Table 1, Figure 1G, 1I). Interestingly, *E(bx)* (also called NURF301) encodes a transcription coactivator that belongs to the ISWI chromatin remodeler complex, another TrxG complex, and negatively regulates the JAK-STAT pathway [26], which is known to interact with *gcm* [27].

We then tested members of two TrxG complexes that specifically counteract Pc function. Trx is a SET-domain containing protein able to induce H3K4 methylation [28]. It has been purified as a subunit of the *Drosophila* COMPASS-like complex [29] and of the TAC1 complex that combines histone methylating and acetylating activities (reviewed in [30]). The *trx* null mutation acts as a suppressor of the *gcm^Pyx^* phenotype (Figure 1G, 1I). Ash1 is a SET-domain protein reported to have histone methyltransferase activity [30]: its null mutation also suppresses the *gcm^Pyx^* phenotype (Figure 1G, 1I). Finally, the *Drosophila* CREBS-binding protein (dCBP) encoded by *nejire (nej)* is responsible for H3K27 acetylation [31] and is associated with both TAC1 and ASH1 complexes. The *nej* null mutation suppresses the *gcm^Pyx^* phenotype (Figure 1G, 1I). In conclusion, we found that mutations in TrxG proteins known to specifically counteract PcG function [12] act as suppressors of the *gcm^Pyx^* phenotype, whereas TrxG members found in chromatin remodeling complexes that are involved in more general transcriptional regulation act as enhancers. We therefore tested members of the two PcG complexes, PRC1 (*Pc*) (three null alleles) and PRC2 (*(esc)*, *E(z)*), as well as the PcG protein recruiter *pipsqueak (psq)*. Mutations in all four genes enhance the *gcm^Pyx^* phenotype (Figure 1H, 1I). Thus, *PcG* mutations act in the same way as mutations in the *TrxG* genes *brm* and *osa*, but have opposing effects compared to mutations in the *TrxG* genes *Ash1*, *trx and nej*. This suggests that a balanced action of these chromatin modifiers regulate *gcm* function.

In sum, the screen allowed the identification of several chromatin factors as *gcm* genetic interactors.

### The Pc protein binds to the *gcm* promoter region

**Figure 2:**
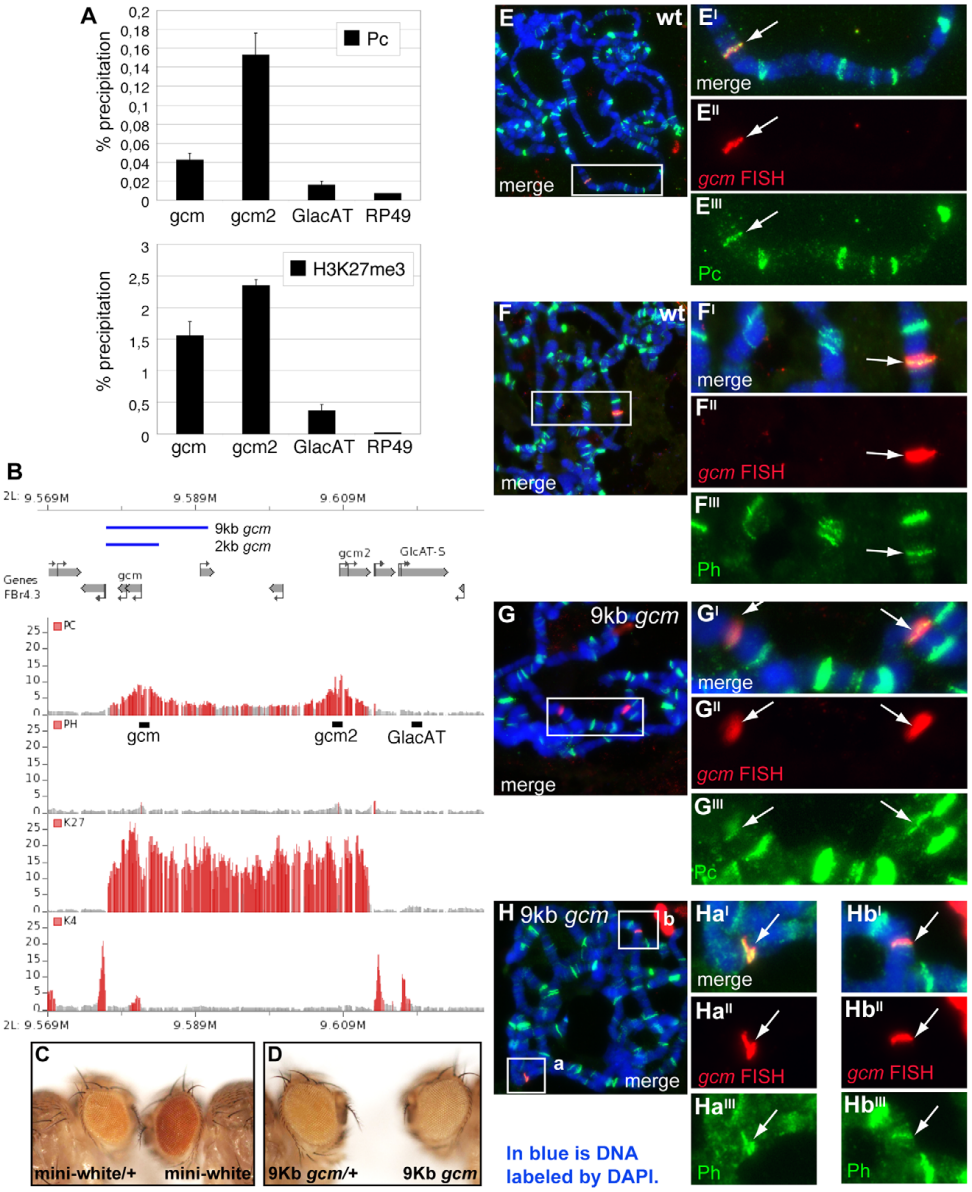
Pc binds to the *gcm* promoter region. (A–B) Association of the *gcm* and *gcm2* loci with PcG proteins. (A) Levels of Polycomb (Pc) binding and H3K27me3 at the *gcm* or *gcm2* gene locus and control regions (GlacAT and Rp49) in *Drosophila* embryos were determined by quantitative ChIP (qChIP) experiments. Results are represented as percentage of input chromatin precipitated. The standard deviation was calculated from two independent experiments. (B) Organization of the *gcm-gcm2* loci, extent of the used transgenic constructs (blue lines) and ChIP-on-chip binding profiles of indicated PcG proteins and histone marks in *Drosophila* embryos. Data were extracted from [33]. The plots show the ratios (fold change) of specific IP versus mock IP assays. Significantly enriched fragments (P-value<1×10^−4^) are shown in red. Black bars indicate the location of primers used for qChIP analysis. (C,D) Eyes from flies carrying an empty mini-*w^+^* transgenic vector (C) or a mini-*w^+^* vector including a 9 kb *gcm* transgene (D). Flies heterozygous for the transgene are on the left, homozygous ones on the right. (E–H) Polytene chromosome immuno-FISH experiments performed on the *gcm* locus and PcG proteins. Immuno-FISH staining in wt (*w^1118^*) flies (E,F) or flies carrying a transgene including a 9 kb region upstream of the *gcm* TSS (G,H), with anti-Pc (E,G) or anti-Ph (F,H) antibodies. Nuclear DAPI labeling in blue. Right panels show higher magnifications of the inserts. Double labeling (E,F) with a *gcm* probe (E″,F″) and anti-Pc antibody (E′″) or anti-Ph antibodies (F′″) detects colocalization (arrow) at one Pc or Ph binding site in wt; transgenic animals (G–H) show a second site of colocalization. (G″–G′″, H-Hb′″). Colocalization of *gcm* and Ph (arrow) in wt (D) and in the transgenic line (F).

*gcm* was identified as a putative Pc target in genome-wide chromatin immunoprecipitation (ChIP) studies on *Drosophila* embryos and different cell lines [32]–[34], we therefore focused on this chromatin factor. As seen in Figure 2B, a Polycomb Response Element (PRE) is present around the transcription start sites (TSS) of *gcm* and *gcm2*, which are organized head to head in a 30 kb region [35]. PRC1 binding at the TSS is accompanied by the H3K27 methylation mark (H3K27me3), the profile of which is much broader, extending throughout the *gcm-gcm2* 5′ regulatory region. As expected, the profile of H3K4methylation complements that of H3K27me3 (Figure 2B). Pc binding was further validated and quantified by qChIP analysis on specific regions including the TSS region for each gene (*gcm*, *gcm2*), or an adjacent region (*GlacAT*) and a negative control (Rp49) (Figure 2A, 2B).

We then asked whether the upstream region of the *gcm* gene bound by PcG proteins is able to recruit PcG proteins in transgenic assays. For this, we examined PcG binding to a transgene containing the upstream region of the *gcm* locus on salivary gland chromosomes by Immuno-FISH experiments. Similar to the endogenous *gcm* locus, which associates with both Pc and Ph proteins (Figure 2E–2F′″), a transgene carrying a *gcm* construct including 9 kb from the promoter region (9 kb *gcm*) induces the recruitment of PcG proteins to an ectopic site (Figure 2B, 2G–2Hb′″). Interestingly, a transgene carrying a shorter construct (2 kb *gcm*) is not able to efficiently recruit PcG proteins (Figure 2B, Figure S3). Importantly, this shorter construct triggers very limited rescue when reintroduced in *gcm* mutant embryos, whereas the 9 kb *gcm* construct rescues the embryonic mutant phenotype almost completely [8], suggesting a correlation between Pc binding and transgene activity. Of note, the transgenes do not contain *gcm2*, excluding the requirement of a gene complex for Pc binding. Moreover, *gcm2* plays a minor role in gliogenesis and its mutation is viable [35] allowing us to focus on *gcm*.

Finally, we tested the 9 kb construct for pairing sensitive silencing (PSS), as transgenes carrying PREs/TREs in *Drosophila* have been shown to share this property ([36], [37]). Transgenic flies carrying the *mini-white* gene typically have eye colors ranging from yellow to orange in a *white* mutant background. Normally, flies that are homozygous for such a transgene have a darker eye color than heterozygotes, as the genetic dose of *mini-white* is doubled. However, with transgenes carrying PRE/TREs, the eye color is similar in homozygotes and heterozygotes or even darker in the latter. This is what we also observed in our transgenic lines (Figure 2C–2D).

Altogether, these data indicate that the *gcm* promoter region contains a PRE and suggest that PcG proteins directly regulate *gcm* expression.

### Reducing the dose of Pc rescues the *gcm* fate conversion phenotype

We next scored for *Pc gcm* interaction in a physiological asset, i.e., in loss of function conditions for both genes. The *gcm-Gal4* line, an insertion in the *gcm* locus, is a hypomorphic semiviable allele in homozygous conditions and can be used to follow *gcm* activation and glial cells using a *UAS-green fluorescent protein (GFP)* line [38]–[40]. We analyzed the expression of GFP as well as that of an independent glial marker (Repo) and a neuronal marker (Elav) in homozygous *gcm-Gal4,UAS-GFP* (referred to as *gcm-Gal4*) animals and in homozygous *gcm-Gal4* animals that are also heterozygous for *Pc*. As a control, we used heterozygous *gcm-Gal4* animals.

**Figure 3:**
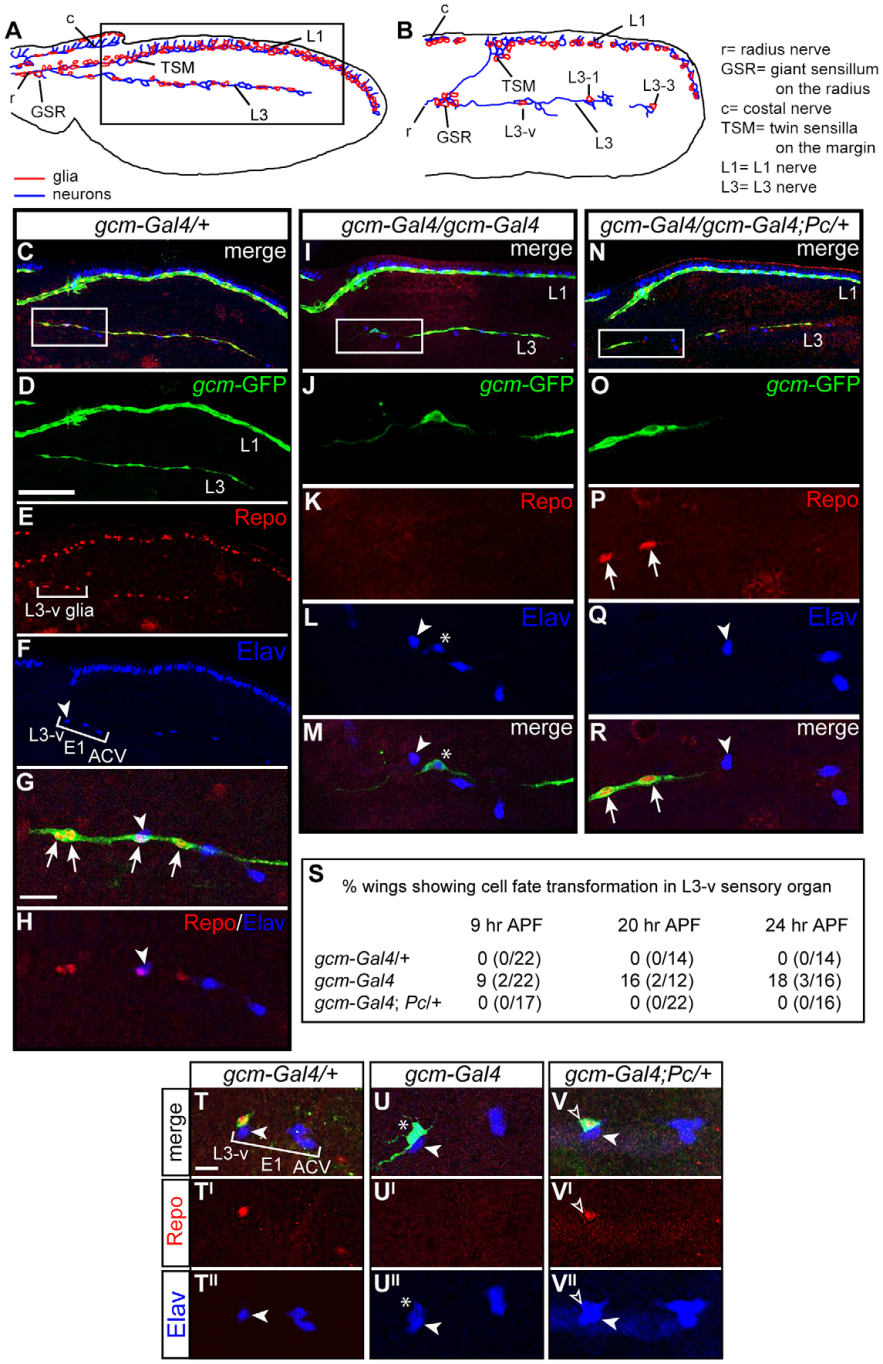
The *Pc* mutation rescues the *gcm* LOF phenotype. (A,B) Schematic drawings showing the pupal wing at (A) 29 and (B) 16 hr APF (in all panels, anterior the top, distal to the right. Inset in (A) indicates the region shown in (C–F,I,N). L3-v, L3-1 and L3-3 indicate the sensory neurons. (C–R) Immunolabeling of 24 hr APF wings: *gcm-Gal4:UAS-GFP/+* (*gcm-Gal4/+*), considered as wt (C–H), *gcm-Gal4* (I–M) and *gcm-Gal4;Pc/+* (N–R). Anti-GFP labeling (green) reflects *gcm* expression, anti-Repo (red) marks glia and anti-Elav (blue) marks neurons. (C–H) Bracket in (E) indicates the glial cells produced by the L3-v sensory organ precursor; bracket in (F) indicates the three proximal neurons (L3-v, ACV, E1). White arrowhead indicates the L3-v neuron. Insets indicate the regions shown at higher magnification (C,I,N). (G,H) The L3-v GP produces several GFP+/Repo+ cells (arrows). In mutant wings (I–M), the L3-v lineage produces only one GFP+ cell (J,M), which does not express Repo (K), but Elav (L,M asterisk indicates the ectopic neuron). In double *gcm* and *Pc* LOF wings (N–R), several GFP+ cells (O,R) express Repo (P) and no ectopic neurons were observed (Q). (S) Quantitative data on the fate transformation phenotype at different stages. (T–V) Immunolabeling in 9 hr APF wings: *gcm-Gal4/+* (T–T″); *gcm-Gal4* (U–U″) and *gcm-Gal4;Pc/+* (V–V″). In all genotypes, one GFP+ cell produced by the L3-v lineage is visible (T,U,V). In the heterozygous wing, this cell expresses Repo (T′) and not Elav (T″). In *gcm-Gal4* (U), the GFP+ cell does not express Repo (U′), but expresses Elav (U″). In the double *gcm* and *Pc* LOF wing, the GFP+ cell (V, empty arrowhead) expresses Repo (V′) and Elav (V′). Scale bars: C–F,I,N = 100 µm; G,H,J–M,O–R,U–W″ = 10 µm.

The *Drosophila* wing contains two major nerves, L1 and L3, covered by glia that depend on *gcm* [41] (Figure 3A, 3B). Because of their simple organization, we focused on the L3 glia, which arise from three SOPs called L3-3, L3-1 and L3-v. Each SOP produces a sensory neuron and a glial precursor (GP) that proliferates and produces four to eight glia that are GFP+ (Figure 3C–3H).

*gcm-Gal4* homozygous flies show the glia to neuron transformation observed in *gcm* null clones [41], albeit at much lower penetrance (Figure 3I–3M). To analyze the phenotype at single cell level, we followed glia from a specific lineage, the L3-v, at the time the GP is generated. At this stage, control L3-v lineages contain a GFP+ cell that expresses Repo and a neuron that expresses Elav (Figure 3S, 3T–3T″). In *gcm-Gal4* homozygous animals, the GFP+ cell expresses Elav rather than Repo (9% penetrance) (Figure 3S, 3U–3U″). By 24 hr after puparium formation (APF), the number of GFP+ and Repo+ cells present in the control animals increases, whereas only one GFP+ cell is present in the transformed lineage, due to lack of proliferation, and this cell is a neuron (Figure 3C–3M). The penetrance of ectopic neurons does not decrease during development (16 and 18% by 20 and 24 hr APF, respectively, Figure 3S), indicating that low Gcm levels trigger a stable fate conversion; a similar phenotype was observed on L1 glia (Kumar and Giangrande, unpublished data).

Based on the genetic data, we then asked whether *Pc* downregulation rescues the phenotype of homozygous *gcm-Gal4* wings. Indeed, no evidence of stable glia to neuron transformation was found in homozygous *gcm-Gal4* wings that carry only one *Pc* functional allele (Figure 3N–3S). The phenotype was verified at early and at late stages of wing development, to exclude the possibility of unstable rescue. These data strongly suggest that Pc affects *gcm* expression in the *gcm-Gal4* line.

### Pc is required for gliogenesis

**Figure 4:**
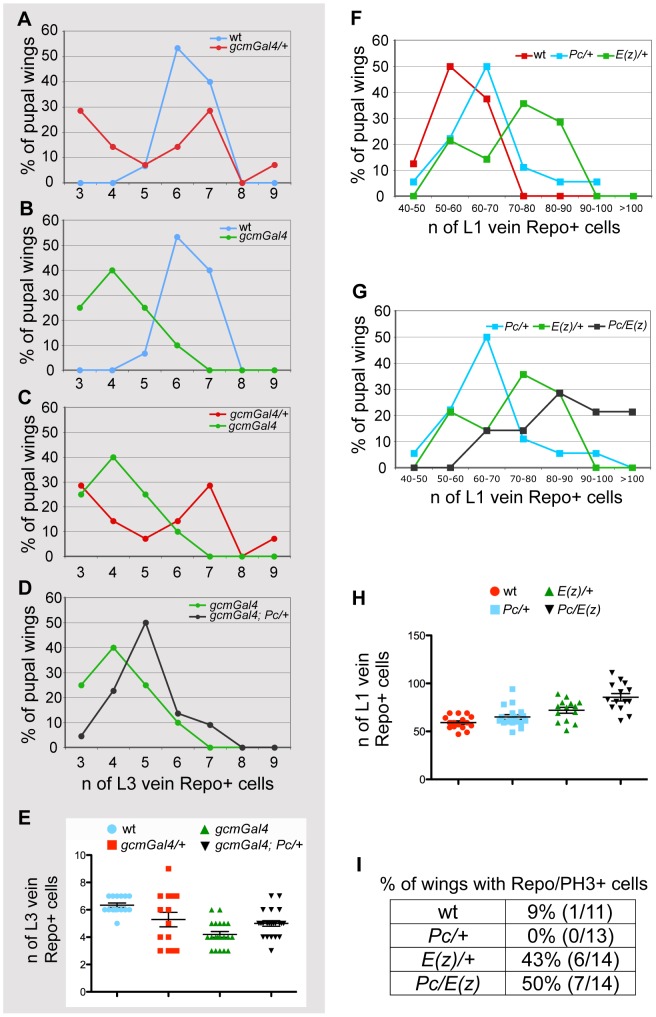
PcG genes control glia proliferation. (A–E) Quantitative analysis of glia at L3 vein position. Graphs comparing animals of different genotypes for the number of glia present on the L3 vein by 20 hr APF. The Y-axis indicates the percentage of wings showing a given number of glia; the X-axis, the number of glia expressing the Repo protein. Color-code is used to distinguish the compared genotypes: (A) wt vs. *gcm-Gal4/+*, (B) wt vs. *gcm-Gal4*, (C) *gcm-Gal/+* vs. *gcm-Gal4*, (D) *gcm-Gal4* vs. *gcm-Gal4*; *Pc/+*. (A) In wild type wings, the number oscillates between 5 and 7, whereas in *gcm-Gal4/+* wings it oscillates between 3 and 9 cells. (B,C) *gcm-Gal4* homozygous animals carry fewer Repo labeled cells and less variation (from 3 to 6) than heterozygous animals (from 3 to 9). This is also reflected by the presence of one peak value for homozygous animals and two for heterozygous animals. (D) Note that *gcm-Gal4*; *Pc/+* animals show an increase of glial cell number compared to that observed in *gcm-Gal4* animals. (E) The graph shows the distribution around the average of the number of Repo+ cells in the different genotypes as indicated by the color code. (F–H) Quantitative analysis of glia at L1 vein position. Graphs comparing animals of different genotypes for the number of glia present on the L1 vein by 24 hr APF. The Y-axis indicates the percentage of wings showing a given number of glia; the X-axis, the quantitative range of Repo expressing cells. Color-code is used to distinguish the compared genotypes: (F) wt vs. *Pc/+* or vs. *E(z)/+*, (G) *Pc/+* vs. *E(z)/+* or vs. *Pc/E(z)*. (F) Most wild type animals show from 50 to 60 glia. (G) Note that most *Pc/E(z)* double heterozygous animals show higher number of glia (from 70 to 80 Repo+ cells) when compared to single heterozygous animals. This is confirmed by more than 20% of wings showing over one hundred Repo+ cells on L1 vein. (H) The graph shows the distribution around the average of the number of Repo+ glia at the L1 vein position in the different genotypes as indicated by the color-code. (I) Quantitative analysis of pupal wings showing a double Repo/PH3+ cell indicating glia proliferation.

In order to extend the above findings, we analyzed late gliogenesis upon lowering the dose of Pc. Differentiated *gcm-Gal4* homozygous wings carry fewer glia than wt wings in which the three glial precursors have divided more than once in most of the cases (Figure S4A, 24 hr APF wings). Given the low penetrance of the fate transformation phenotype, this suggests an additional, later, effect on the glial cell number. To clarify the nature of the phenotype we counted the Repo+ cells just after the first division of the three L3 GPs in *gcm-Gal4* homozygous wings that showed no fate transformation. We could confirm a decreased number of cells (Figure 4B, 4E, S4B, 20 hr APF wings), complementing the finding that sustained *gcm* expression induces glial overproliferation (embryo: [11]; wing: Kumar and Giangrande, unpublished data). Of note, the *gcm-Gal4/+* wings already show a minor but consistent defect as there are cases in which the three GPs have not proliferated yet, which does not occur in wild type wings of the same stage (Figure 4A and 4E, Figure S4B). Moreover, heterozygous wings show a high variance in the number of Repo+ cells. Finally, homozygous *gcm-Gal4* wings expressing a single Pc show a higher number of glia compared to those found in homozygous *gcm-Gal4* wings (Figure 4D and 4E, Figure S4B), confirming that Pc negatively controls Gcm. This was confirmed by the significant P values obtained with different robust non-parametric tests comparing the homozygous wings with the homozygous wings that carry one dose of Pc (Mann Whitney test P = 0,0127; Wilcoxon test P = 0,0122). Moreover, one-way Anova comparison of the three genotypes (*gcm-Gal4/+*, *gcm-Gal4* and *gcm-Gal4*; *Pc/+*) also produces a significant value (0,0028). These data indicate a partial rescue of the *gcm-Gal4* proliferation phenotype by Pc, the moderate difference likely depending on the fact that only one dose of Pc is deleted.

To understand the role of Pc in gliogenesis, we also analyzed *Pc* mutant animals in an otherwise wt background and asked whether the mutation affects the number of glia (Figure 4F–4H, Figure S4C 24 hr APF wings) and the frequency of glial dividing cells (Figure 4I). Since removing Pc completely leads to pleiotropic defects, we used heterozygous *Pc* animals and counted the number of Repo+ cells on the L1 nerve, which shows massive gliogenesis, compared to the sparse glial cells present on the L3 nerve [41]. While the number of Repo+ cells increases very moderately in *Pc/+* compared to wt wings (P = 0,03), a stronger, significant, increase is observed in *E(z)/+* wings (P = 0,0007), which have a compromised PRC2, and an even stronger phenotype is observed in double heterozygous *Pc/E(z)* animals (P = 3,9×10^−6^), which display compromised PRC2 and PRC1 (Figure 4F–4H, Figure S4C). Finally, we labeled wings with Repo and phospho-histone H3 (PH3) as a mitotic marker. By 24 hr APF, the Repo/PH3+ cells are very rare in wt wings (1 Repo-PH3+ cell in 1/11 wings) (Figure 4I). *E(z)/+* or *Pc/E(z)* double heterozygous animals, which show the most significant increase in glial cell number, show a significant increase in the number of wings with proliferating glia, whereas *Pc/+* animals, in which the increase in glial cell number very small, do not. Thus, PcG proteins likely synergize and affect both glial differentiation and proliferation.

### Pc represses the maintenance of *gcm* expression

We next analyzed the role of Pc on the *gcm* expression profile. Positional cues first trigger initiation of transcription, then Gcm positively autoregulates [7] and, as the glial fate is established, *gcm* expression progressively decreases so that its transcripts are no longer present in mature glia [42]. We analyzed the initiation of *gcm* transcription in *gcm-Gal4/+*; *Pc/+* wings. Previous analyses showed that the *gcm* RNA becomes detectable by 8–9 hr APF (Van de Bor and Giangrande, unpublished data). We therefore analyzed 7–8 hr APF wings and found that the GFP appears at the same time as in wt animals (data not shown). Since the binary Gal4 system may not faithfully reproduce the temporal pattern, we analyzed wings carrying one dose of Pc and the P-mediated insertional *gcm^rA87^* allele expressing the LacZ reporter and confirmed that the β-Gal labeling starts as in wt animals (Figure S5). The finding that Pc does not affect initiation of *gcm* expression is in line with the wt number of GFP+ cells observed in homozygous *gcm-Gal4* wings at early stages, even in cases in which glial cells convert into neurons.

**Figure 5:**
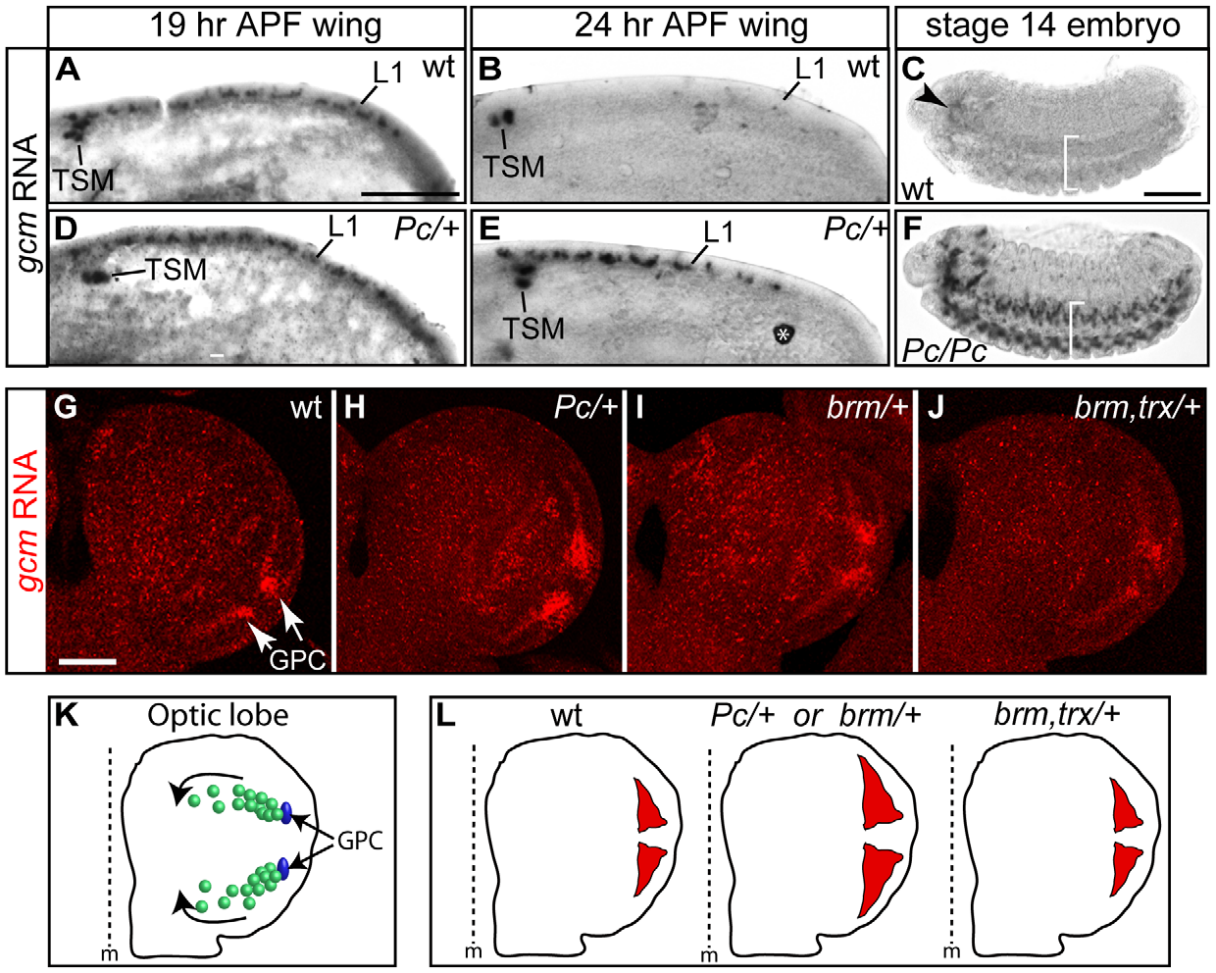
*gcm* is overexpressed in *Pc* mutants. (A–J) *In situ* hybridization with a *gcm*-specific probe. (A,B,D,E) 19 hr APF wings: *gcm* is expressed at the L1 nerve position (L1) and in the so-called twin sensilla of the margin (TSM) in wt (A) as well as in *Pc/+* animals (D); by 24 hr APF, *gcm* is no more expressed in wt (B), but persists in *Pc/+* wings (E) (asterisk indicates a non-specific signal). (C,F) *gcm* expression in the embryonic brain (arrowhead) and in the ventral cord (brackets) fades by stage 14 in wt (C), but persists in *Pc* mutants (F) (lateral views, anterior to the left). (G–J) optic lobe partial projection (anterior to the top; scale bar = 100 µm): in wt (G), *gcm* is expressed at the position of the lamina glial cell precursor (GPC) area (arrows); *gcm* expression in *Pc/+* (H), in *brm/+* (I) and in *brm*, *trx/+* double mutants (J). Note that we focused on early third instar larvae, when the first burst of expression takes place in the GPC region. At that time, *gcm* is just starting being expressed in the other territories that have been previously described as *gcm* positive [39], [40], [43]. (K) Schematic representation of optic globe *gcm*-dependent lamina glial lineages. In blue, the GPCs. In green, differentiating and migrating glial cells (direction shown by the arrows). (L) Schematic representation of the areas of *gcm* expression (red) in the GPC region, based on the above *in situ* analyses.

We also performed in situ hybridization with a *gcm*-specific probe in *Pc/+* wings. We took advantage of the supernumerary glia phenotype to see whether Pc helps repressing the maintenance of *gcm* expression. *gcm* transcripts are well visible on both wt and *Pc/+* wings by 19 hr APF, a stage at which the glial precursors have differentiated (Figure 5A, 5D). By 24 hr APF, however, they are absent in wt, but still present in *Pc/+* wings (Figure 5B, 5E), which correlates with the slight increase in glial number observed in *Pc/+* animals. Interestingly, *Pc/+* wings do not show *gcm* expression at ectopic positions, suggesting that the absence of *Pc* induces a failure in repressing *gcm* maintenance rather than a global loss of silencing in whole tissues.

We extended the data by analyzing other stages and tissues. In the brain, gcm is expressed in several cell populations: GPC and its glial progeny, lamina neurons, central brain neurons and medulla glia [39], [40], [43], [44]. We focused on *gcm* expression at the position of lamina glial precursors (GPCs), which produce numerous cells that migrate and form the glia of the lamina visual ganglion (Figure 5K) [39], [40], [43]. For the sake of simplicity, we analyzed the optic lobes at a stage at which *gcm* is detectable in the GPC area but just starts being expressed in the other regions. In wt animals, *gcm* expression fades away as glia differentiate and migrate (Figure 5G, 5K, 5L), whereas in *Pc/+* animals *gcm* is expressed in an expanded area (Figure 5H, 5L). Moreover, *gcm* is overexpressed in *brm/+* brains and this phenotype is suppressed in *brm,trx*/+ animals. This shows that *brm* acts similar to *Pc* on *gcm* expression, and both act antagonistically to *trx*, in line with the genetic data (Figure 5H–5J, 5L). All the phenotypes were quantified by comparing the intensity and the area of the *gcm* signal (see Text S1, Figure S4E). In the double mutant, the area of labeling resembles that observed in wt animals and the intensity of the signal is even lower than that observed in wt animals. Future analyses will determine whether the increase of *gcm* expression in the mutant backgrounds reflects longer perdurance in migrating glia, production of more glia or production of more glial progenitor cells in the larval lamina. In some preparations, labeling in other regions is also observed, depending on sample orientation. Even though we cannot formally exclude the possibility that this represents ectopic labeling, these regions correspond to the other positions at which *gcm* accumulates at slightly later stages in wild type animals, suggesting that in those regions as well Pc negatively controls *gcm* expression.

Finally, we analyzed *gcm* transcripts in *Pc* embryos. In wt animals, *gcm* is expressed at early stages of glial development and transcripts subsequently fade away, first in the ventral cord and then in the brain [42]. The most frequent phenotype of *Pc* mutant embryos is a persisting *gcm* expression in the brain, but we also found extreme cases of late *gcm* expression in the ventral cord (Figure 5C, 5F). The embryonic and the postembryonic brains contain too compact and numerous glia and the perdurance in the ventral cord is a rare event, likely due to the Pc maternal component.

Although these tissues/stages do not allow quantitative analyses of glial cells, the expression data and the wing phenotype strongly suggest that Pc represses *gcm* maintenance. Altogether, our observations highlight the importance of Pc in tightly regulating Gcm levels.

### *Pc* represses *gcm* positive autoregulation and a downstream *gcm* target

**Figure 6:**
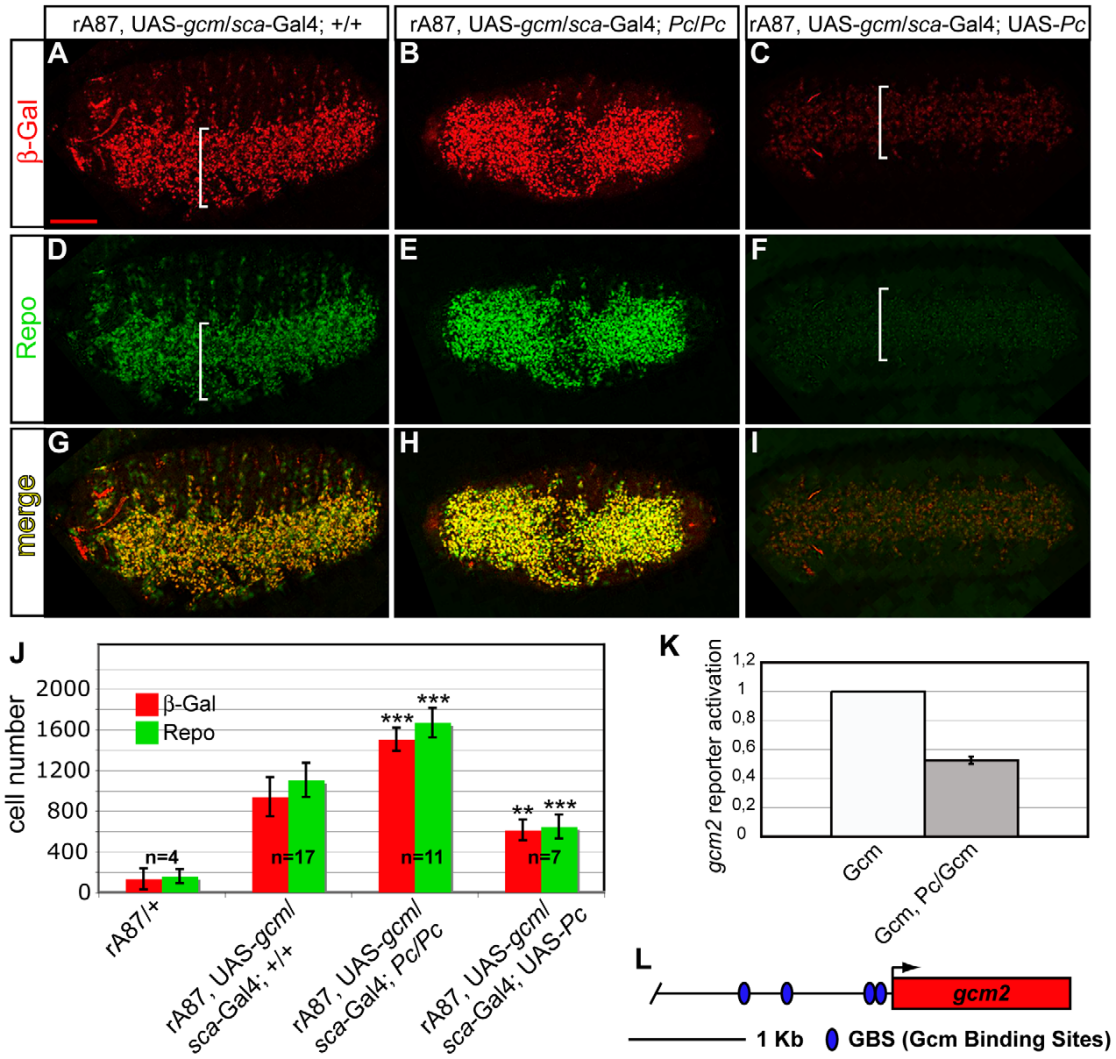
*Pc* inhibits *gcm* autoregulation and glial differentiation. (A–I) Immunolabeling of *gcm* GOF embryos carrying *rA87*, a lacZ insertion that detects endogenous *gcm* expression, *UAS-gcm* and the *scabrous-Gal4* driver, active in the whole embryonic ventral cord (white brackets) (A,D,G); *gcm* GOF, *Pc* LOF embryo (B,E,H); *gcm* GOF, *Pc* GOF embryo (C,F,I). Ventral views. *gcm* GOF causes endogenous *gcm* overexpression (A), and ectopic glial cell production (D,G). In a *Pc* LOF embryo, the number of ß-Gal+ cells increases (B), as well as the number of Repo+ (E,H); in a *Pc* GOF embryo, the number of ß-Gal+ (C) and Repo+ (F) cells decreases. (J) Histograms present the average number of ß-Gal+ (red) and Repo+ (green) cells in embryonic thoracic segments (y-axis) from different genotypes (x-axis). *P-*values of t- test vs. *gcm* GOF: *gcm* GOF *Pc* LOF (ß-Gal 1,7×10^−5^; Repo 1,5×10^−5^); *gcm* GOF *Pc* GOF (ß-Gal 0,003; Repo 0,0001). Scale bar = 100 µm. The graph (K) shows the activation of a 2 kb *gcm2* promoter reporter construct displaying four GBSs (L). The ratio between reporter activity upon Gcm/Pc coexpression vs. that observed upon Gcm expression alone indicates that the *gcm2* promoter is activated when Gcm is expressed in S2 cells and repressed upon Gcm and Pc coexpression.

To assess whether Pc directly represses *gcm*, we used in vivo and in vitro assays. Gcm directly and positively autoregulates and alteration of this feedback loop severely affects its gliogenic potential, providing further evidence for the importance of Gcm maintenance at a precise developmental time [7], [9]. In vivo autoregulation can be documented in gain of function experiments by using the *gcm^rA87^* allele. We asked whether Pc negatively controls Gcm autoregulation by comparing animals that simultaneously overexpress Gcm and Pc to control animals that only overexpress Gcm. Compared to controls, Pc and Gcm cooverexpressing embryos show a drastic reduction in the number of β-Gal+ cells as well in the intensity of β-Gal labeling (Figure 6A, 6C). Accordingly, co-overexpression reduces the number of ectopic glia as assessed by the Repo marker (Figure 6D, 6F, 6G, 6I). Moreover, and in line with these results, overexpressing Gcm in *Pc* loss of function embryos triggers a significant increase in the number of autoregulating cells compared to that observed in control animals (Figure 6A, 6B). Accordingly, these animals show an increased number of ectopic Repo+ cells (Figure 6D, 6E, 6G, 6H). These data were quantified upon counting the number of β-Gal+ and Repo+ cells (Figure 6J). Loss and gain of function of Pc do not, on their own, alter the expression of the Repo marker (Figure S6).

To evaluate whether the inhibitory effects of Pc in the Gcm pathway are direct, we used transactivation assays in which we transfected S2 cells with a Gcm expression vector and a reporter of its activity in presence or in absence of a Pc expression vector.

We first analyzed the *repo* promoter, a major direct Gcm target that contains several Glide Binding Sites (GBSs) [45] (Figure S7D). This promoter is inactive in S2 cells, but Gcm expression is sufficient to activate it. Upon cotransfection with Gcm and Pc expression vectors, however, the transactivation induced by Gcm decreases significantly (Figure S7C, S7D). We repeated the same type of assay using a second, transiently expressed, promoter depending on *gcm*. The *gcm2* 2 kb proximal promoter contains four GBSs and was previously shown to be activated by Gcm in transfection assays [35] (Figure 6K, 6L), more robustly than the *gcm* 2 kb promoter itself, which only contains one GBS. As for *repo*, the cotransfection with Gcm and Pc reduces the activation of the *gcm2* promoter. Thus, Pc represses the expression of Gcm stably and transiently expressed targets.

In sum, the above data support the hypothesis that *Pc* represses *gcm* autoregulation and Gcm downstream targets, thereby inhibiting glial development.

## Discussion

Cell fate determination and maintenance require pathways that finely modulate gene expression and hence ensure the proper balance of cell types in metazoa. The pleiotropic and genome-wide effects of such pathways still hamper clear understanding of their impact and mode of action at single cell level. Our screen and genetic analyses in the *Drosophila* model unveil the role of the Polycomb chromatin modifier in the generation of glial cells upon fine modulation of the transiently expressed fate determinant *gcm*.

### A genetic screen that identifies novel *gcm* interactors

The genetic screen over a sensitized background proved to be an extremely sensitive tool, as it allowed us to identify several genes that in heterozygous conditions are able to modify the strong dominant *gcm^Pyx^* phenotype. The screen also provided hints onto the function of the interactors, suppressors or enhancers of a given phenotype. For example, *sna* and *esg* act as *gcm^Pyx^* suppressors, in line with the fact that *gcm^Pyx^* triggers the expression of NB-specific genes [20]. Identifying an interactor provided an entry point to find members of the same pathway that were initially underscored because located in deficiencies with moderate phenotypes (perhaps due to the presence of genes with opposite effects) or in regions that were not covered by the deficiencies. In the first case is *Pc*, in the second are *osa* and *Ash1* (Figure S2). The screen also identified members of other signaling pathways (Table 1, Figure S2). One of them depends on *Notch (N)*, which controls *gcm* expression [20]. While the used Deficiency kit does not cover *N* itself, we identified Suppressor of Hairless (Su(H)), which regulates the transcription of the N targets, and Lethal (2) giant disc 1, which negatively regulates N receptor trafficking ([46] and references therein). We also tested and validated the genetic interaction with other members of the cascade, including N, its ligand Delta, one of its targets, Enhancer of split, and Groucho, a transcriptional repressor and a partner of Su(H). Future studies will dissect the role of this and of the other pathways on the Gcm cascade.

### *gcm* genetically interacts with TrxG proteins

Several TrxG proteins act as genetic modifiers of the *gcm^Pyx^* phenotype. TrxG proteins were initially identified as positive regulators of HOX genes and considered as PcG counteractors. In recent years, however, it has become evident that they have a much wider role in gene regulation and it is unclear whether they mainly antagonize PcG functions or whether they globally control gene expression [12]. Interestingly, the three TrxG proteins that behave as positive regulators, Trx, Ash1 and dCBP, are found in TAC and ASH1 complexes that contain a histone acetylation activity. The dCBP histone acetyltransferase present in these complexes acetylates H3K27, a modification that is associated with PcG target genes when they are active [34]. This modification is incompatible with Pc dependent H3K27me3, as these modifications occur on the same amino acid. Thus, Trx- and Ash1-associated dCBP might be a key player in counteracting PcG-dependent silencing of the *gcm* gene [31]. Future studies will address the role of dCBP onto the Gcm cascade.

*osa* and *brm* act as negative regulators of *gcm*. TrxG proteins can form different complexes that have distinct properties and in some instances repress gene expression. For example, Trx and Brm, which belong to different molecular complexes [30], act positively on the HOX genes and influence a homeotic transformation phenotype in the same way [47], however, Brm-containing complexes mediate transcriptional repression of genes other than the HOX genes [48]. The emerging view is that the SWI/SNF TrxG proteins act as transcriptional activators or repressors depending on the temporal and spatial context [49]. Further studies will determine whether the TrxG proteins acting as negative regulators of *gcm* directly repress its expression or induce a *gcm* repressor.

### Pc modulates the transient expression of the fate determinant *gcm*

PcG proteins repress homeotic genes to ensure the maintenance of transcriptional states and provide a cellular memory that is transmitted upon cell division, in contrast, their mode of action in the control of more dynamic processes remains elusive. We show in vivo that members of the PcG negatively regulate the *gcm* pathway during glial fate establishment and proliferation. At least in the first step, a process based on cell memory can be excluded, as Pc acts prior to the division of the GP, the cell in which *gcm* starts being expressed [41].

The qChIP assay as well as the expression, the S2 cell transfection and the autoregulation data strongly suggest that Pc directly represses *gcm* transcription maintenance. In addition, the phenotypes observed upon changing the relative gene dosage indicate that Pc and *gcm* need to be present in appropriate amounts. The importance of an adequate balance between positive (Gcm) and negative (Pc) factors in the establishment of the glial fate is also provided by a rare phenotype observed in a *gcm-Gal4*; *Pc/+* background (1/17 wings) in which the GFP+ cell expresses Repo and Elav, indicating an intermediate glial/neuronal state (Figure 3V–3V″). Thus, Pc acts by finely tuning a transiently expressed fate determinant.

**Figure 7:**
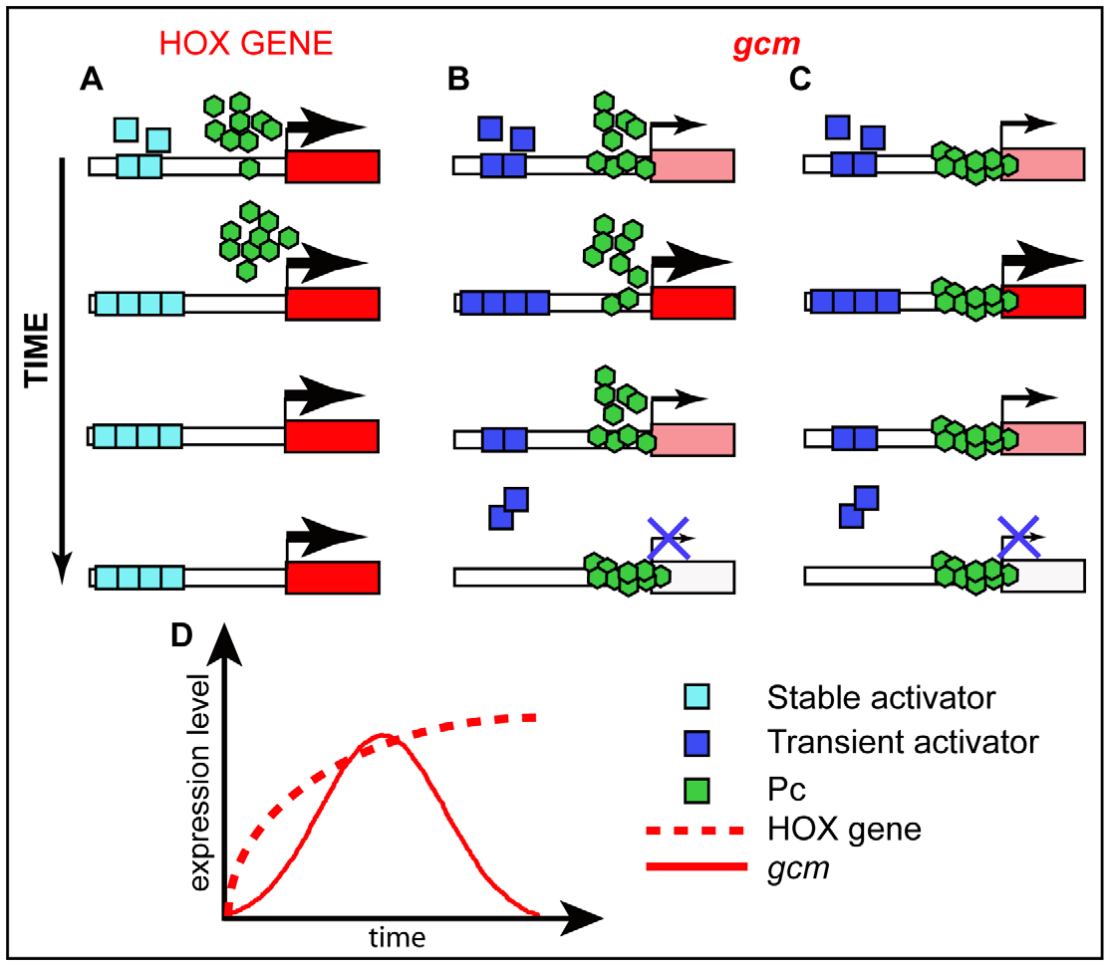
Schematic models for Pc mode of action. (A–C) Possible mode of action of Pc on HOX genes and on a developmental gene that is transiently expressed. See the different expression profiles on the schematic graph (D). (A) promoters that are constantly active (HOX) are devoid of Pc binding. (B) Transient activator(s) may compete with PcG proteins for binding thereby modulating the levels of expression of dynamically expressed promoters. (C) Active, dynamically expressed, promoters may be constantly occupied by PcG proteins and their expression levels depend on the amount of transient activator(s) available and bound to the promoter. Color code legend is included.

We speculate that the role and the mode of action of chromatin factors depend on the target. HOX promoters, which require to stay in an ON or OFF state, may involve strong binding/high accumulation of chromatin regulators and several studies have already shown that HOX activators drastically reduce K27me3 and also PcG protein binding (Figure 7A) [34], [50], [51]. More dynamically expressed genes may involve less strong binding, a configuration that allows modulation of gene expression. From a mechanistic point of view, as the activator of the transiently expressed genes disappears, PcG proteins may gradually bind and turn these genes OFF (Figure 7B) although we cannot formally exclude that PcG proteins may simply provide a constant repressive background as a threshold for activation (Figure 7C).

In line with these hypotheses, HOX and Gcm display different behaviors. A fragment of 219bp from Fab7, the classical PRE described on a HOX promoter, is sufficient to recruit PcG proteins on salivary glands [52], whereas a 2 kb *gcm* carrying the PRE seems very inefficient. In addition, the intensity of Pc, Ph and ‘recruiters’ peaks onto the *gcm* promoter is very low, definitely weaker compared to those found on the classical HOX PRE (Figure S8). Finally, the heterozygous *Pc/+* mutation only temporarily prolongs *gcm* expression (Figure S5I), whereas it produces a long lasting HOX-dependent phenotype [53], [54].

Understanding the precise molecular events will require the development of new tools and the in vivo analysis of chromatin organization at the level of specific cell types or in single cells. Our data nevertheless clearly show that Gcm and Pc compete with each other: PcG proteins bind *gcm* genes as well as *repo* (Figure 2, Figure S7, Figure S8) [33] and counteract Gcm activity. We therefore speculate that Gcm displaces Pc from its target promoters, including itself, which would explain how a general chromatin regulator impinges onto a cell-specific transcriptional program. In mammals as well it has been suggested that cell fate transcription factors play a role in PcG recruitment and displacement and some of them were shown to be PcG targets ([55] and reference therein). Finally, 63 genes are common Pc *and* Gcm targets, as revealed by analyzing the Pc binding sites in embryos and in cell lines (from [33] and [34]) and the genes positively regulated by Gcm identified by microarray (from [23]). Clearly, genome-wide screens for direct Gcm targets will be necessary to support the hypothesis of Pc displacement. These studies will also assess whether the impact of the PRCs on glial proliferation is direct or mediated by Gcm.

### Pc represses gliogenesis

The rescue of the *gcm*-dependent phenotype upon Pc downregulation indicates a role for this chromatin factor in glial repression. Interestingly, upregulating or downregulating Pc does not per se produce the opposite fate transformation (Figure S4D), whereas it does modify the number of glia, showing that distinct protein levels are required in different processes. In vertebrates, the PRC2 is also involved in the production of glial cells, which differentiate after a wave of neurogenesis. However, different results were obtained depending on the experimental asset. Livesey and collaborators ([56]) deleted Ezh2 constitutively, thereby altering the balance between self-renewal and differentiation, and found precocious astrocyte differentiation. In contrast, Gotoh and collaborators [57] used a conditional Ezh2 knockout and documented a decrease in astrocyte differentiation. In the first case, the authors speculated that the altered timing of neurogenesis and accelerated onset of gliogenesis are secondary to the primary function of PRC2 in cortical progenitor cells. In the second report, it was shown that Ezh2 represses Neurogenin1, which controls timing during corticogenesis and therefore the relative production of neurons and glia. While these studies indicate the importance of chromatin modifiers in the nervous system, they do not clarify the role of PRCs in gliogenesis. In our study, the combined use of sensitive tools demonstrates that the Pc chromatin factor directly inhibits gliogenesis and identifies *gcm* as a major target in the pathway. First, we used sensitized backgrounds rather than total knockouts, which makes it possible to score for subtle phenotypes. Second, we analyzed the mutants at the single cell resolution and therefore scored for direct, cell autonomous, effects of the *Pc* mutation. Third, we analyzed a gene that plays an instructive role rather than simply being permissive for gliogenesis. Fourth, *gcm* carries a functional PRE and competes with Pc on its targets. Altogether, these findings reinforce the view that distinct chromatin states characterize specific cell fates, as also illustrated by the low levels of histone acetylation observed in both fly and vertebrate glia [6], [58].

## Materials and Methods

### *Drosophila* stocks and crosses

Flies were grown on standard cornmeal/molasses medium at 25°C. The deficiency kit was obtained from the Bloomington Stock Center (Bloomington, IN), see Supplementary Material and Methods.

### Bristle phenotype analysis

For the qualitative screen: for each cross (180 deficiencies), double heterozygous females carrying the *gcm^Pyx^* allele and a deficiency were scored for the supernumerary bristle phenotype and compared to sibling females carrying the *gcm^Pyx^* allele and the balancer from the deficiency stock. This allowed us to classify each deficiency as *gcm^Pyx^* modifier or not modifier (Figure S1Aa). 75 deficiencies covering 42 genomic regions were selected for quantitative analyses (Figure S1Ab); for each genotype we counted the bristles from 10–80 heminota. The flow chart in Figure S1A shows the details of the screen. Average values +/− SEM were calculated and, for genotype comparisons, the statistical significance was estimated by t-test.

To overexpress *esg* or *osa*, respectively, *w*; *EP(2)0684/CyO* or *w*; *P{w[+mC] = UAS-osa}s2/CyO* females were crossed with *w*; *gcm^Pyx^/Sp*; *hs-Gal4/Sb* males. A 30 minute heat-shock pulse on 2nd instar larvae was performed at 37°C.

### qChIP

qChIP was performed as in [33]. Primers are listed in Figure S3A.

### Immunolabeling and *in situ* hybridization

these assays were performed as in [41] and [44]. For the antibody list as well as for the protocol of wing and embryo mounting and analysis by confocal microscopy, see Text S1. Repo and β-Gal positive cells from embryonic VC were subjected to quantification in 3D image using Imaris 7.2 software. Masks were generated as a region of interest for three thoracic segments along the z-stack, then volume image was visualized and the “crop 3D” function was applied to isolate the region of interest. Voxels (volume picture element) corresponding to cells were identified based on size and intensity. Then automatic voxel (cell) counting was performed in the region of interest. t-test was used to quantify the difference between genotypes. For immuno-FISH staining on polytene chromosomes [59], three consequent probes covering around 3 kb around *gcm* TSS were used, see Figure S3. Unless specified, all quantitative analyses used the t-test.

### Cell transfection and reporter activation assay

The *gcm2* promoter construct is *pBLCAT6-1.96* from [35]. The 4,3 kb of the *repo* promoter [45] was cloned into the pRed H-Stinger vector (Berzsenyi and Giangrande, unpublished data). *pPAC-gcm* is described in [7]. *UAS-gcm* is described in [42]. *pPAC-Pc* and *UAS-Pc* were obtained by cloning the entire *Pc* cDNA in backbone vectors. *pPAC-lacZ* was a gift from T. Cook. Transient transfection of *Drosophila* S2 cells [60] was performed using Effectene (Qiagen) according to the manufacturer's instructions using 3 µg of total DNA. For CAT assay to evaluate the activation of the 2 kb *gcm2* reporter construct (*pBLCAT6-1.96*), cells were harvested 48 hr after transfection and normalized for ß-Gal activity. CAT levels were determined using the CAT ELISA kit (Roche). For *repoRFP*, images of cells were acquired 48 hr after transfection, and the green (*UAS-GFP*)/red (*repoRFP*) cells, were quantified automatically using the ImageJ software.

## Supporting Information

Figure S1 Genetic screen for *gcm^Pyx^* modifiers and validation of candidate genes. (A) Flow-chart of the screen: *gcm^Pyx^/CyO*, *twist-LacZ* flies were crossed to Bloomington Deficiency kit strains. The bristle phenotype was compared between sibs: control (*gcm^Pyx^*/Balancer) and experimental females (*gcm^Pyx^*/*Df*). The screen was performed in three steps (primary qualitative, primary quantitative, secondary) and followed by gene validation. The number of analyzed deficiencies and the quantitative data are presented. Bal = balancer, Dfs = Deficiencies. (B) Primary quantitative screen deficiencies summary. Top: chromosome arms, names and cytology of deficiencies selected as strong modifiers of the bristle phenotype: suppressors (S) and enhancers (E). Bottom: total number of modifier deficiencies on each chromosome arm, number of suppressor and enhancer deficiencies. (C) Histograms present the average number of bristles per heminotum (y-axis) in different genotypes (x-axis). Large (*Df(2L)TE35BC-24*) and small *Df(2L)ED1050*) deficiencies cover *esg*, *wor* and *sna* genes. *Pyx/esg* GOF stands for *gcm^Pyx^*/EP(2)*0684*; *hs-Gal4*. Phenotype observed upon heat shocking *gcm^Pyx^* animals that carry the *hs-Gal4* driver and the *EP(2)0684* insertion expressing *esg* in response to Gal4 induction. Note that both deficiencies eliminate *wor*, *esg* and *sna*, but only the large one covers the *Su(H)* mutation, which acts as suppressor. This may explain why the large deficiency seems to act as a stronger suppressor. (C) Deficiencies deleting *pim* and *crol* genes as well as their single mutations. In each graph, average values are indicated +/− SEM (bars); *P-*values from t-test are indicated in the following way: *** (*P*≤10^−3^), ** (*P*≤10^−2^), * (P≤5×10^−2^). *P-*values vs. *gcm^Pyx^*/+: *gcm^Pyx^*/Df(2L)TE35BC-24 (9,3×10^−7^); *gcm^Pyx^*/Df(2L)ED1050 (4,7×10^−8^); *gcm^Pyx^*/*esg* (5,4×10^−6^); *gcm^Pyx^*/*sna* (1,3×10^−18^); *Pyx/esg* GOF (0,005). *gcm^Pyx^*/Df(2L)J2 (4,7×10^−8^); *gcm^Pyx^*/*pim* (5×10^−5^); *gcm^Pyx^*/Df(2L)esc-P2-0 (6,8×10^−5^); *gcm^Pyx^*/*crol* (8,3×10^−7^).
(TIF)

Figure S2 Summary of the genes analyzed over *gcm^Pyx^*. From left to right, columns indicate the name of the gene, the cytology, the heterozygous phenotype over *gcm^Pyx^* (No – no effect; S – suppressor; E – enhancer), the phenotype of the large deficiency over *gcm^Pyx^* (nt – the gene region is not covered by the tested deficiencies), the function of the gene, previous identification as a Gcm target/regulator, references. TF = transcription factor, TrxG = Trithorax group, PcG = Polycomb group; JAK-STAT = Janus kinase/Signal Transducer and Activator of Transcription.
(PDF)

Figure S3 The Pc binding region and the polytene chromosomes of the 2 kb transgenic line. (A) List of primers used for immuno-FISH and qCHIP. (B) Immuno-FISH staining (anti-Pc, *gcm*) on polytene chromosomes carrying a transgene including a 2 kb region upstream of the *gcm* transcription start site.
(TIF)

Figure S4 Mutant phenotypes in wings and brains. (A) Percentage of 24 hr APF wings of the described genotypes carrying different numbers of Repo+ cells on the L3 vein. (B,C) Summary tables showing the number of Repo+ cells observed on the L3 (B) and L1 veins (C) in each pupal wing analyzed. The observed minimum and maximum value in samples of the different genotypes are respectively highlighted in pale-blue and red. (D) Number of wings scored for the fate transformation phenotype in heterozygous *Pc/+* wings or in *Pc* overexpressing wings, using two different *Gal4* drivers. (E) Quantitative analysis of *gcm* expression in the optic lobe (see Figure 5J–5H): histograms present the average signal intensity (y-axis) in the different genotypes (x-axis). P values of t-test vs. wt: *Pc/+* (1,7×10^−6^); *brm/+*(0,0009); *brm,trx/+*(0,0008).
(TIF)

Figure S5 Initiation and extinction of *gcm* expression in *Pc/*+ wings. (A–G) Immunolabeling of 7 hr APF wings from the P-mediated insertional *gcm^rA87^* allele expressing the LacZ reporter, anterior to the top, distal to the right. By this stage, the β-Gal labeling is still not present onto the L1 vein in most of the wings (11/12); in one wing (A–D), one β-Gal labeled cell is visible at the distal tip (β-Gal in red, neuronal labeling (Elav) in green). This cell (arrow) is close to a neuron (arrowhead), (D) shows a magnification from the boxed region. L1 and L3 indicate the position of the L1 and L3 veins, respectively. In wings heterozygous for *Pc* (n = 13) (E–G), no precocious β-Gal labeling was observed on the L1 vein. (H,I) *In situ* hybridization with a *gcm*-specific probe on 29 h APF wings from wt (H) and from *Pc/+* (I) animals. Note that, in both backgrounds, *gcm* is no more expressed.
(TIF)

Figure S6 Repo expression in wild type, Pc LOF and GOF. Immunolabeling to show Repo protein in st. 14 embryos. Ventrolateral view in wild type (A), *Pc/Pc* (B), *scabrous-Gal4/UAS-Pc* animals (C).
(TIF)

Figure S7 Pc binds to and acts on the *repo* promoter. (A) Levels of Pc binding and H3K27me3 at the *repo* locus in *Drosophila* embryos were determined by quantitative ChIP (qChIP) experiments, the *bxd* locus was used as a positive control. Results are represented as percentage of input chromatin precipitated. The standard deviation was calculated from two independent experiments. (B) ChIP-on-chip binding profiles of indicated PcG proteins and histone marks in *Drosophila* embryos at the *repo* regulatory region obtained as reported by [33]. The plots show the ratios (fold change) of specific IP versus mock IP assays. Significantly enriched fragments (P-value<1×10^−4^) are shown in red. Black bars indicate the location of primers used for qChIP analysis. The graph (C) shows the activation of a reporter construct carrying 4 kb from the *repo* upstream regulatory sequence displaying eleven GBSs (D). The ratio between reporter activity upon Gcm/Pc coexpression and that observed when only Gcm is expressed indicates that the *repo* promoter is activated when Gcm is expressed in S2 cells and repressed upon Gcm and Pc coexpression.
(TIF)

Figure S8 Comparison between *gcm/gcm2* and bx/*bxd* PREs. ChIP-on-chip binding profiles of indicated PcG proteins and histone marks in *Drosophila* S2 cells from (Schwartz et al., 2006) or *Drosophila* embryos from (Schuettengruber et al., 2009). Nomenclature as in Figure 2.
(TIF)

Text S1 Supplementary materials and methods.
(DOC)

## References

[1] Hosoya T, Takizawa K, Nitta K, Hotta Y (1995) glial cells missing: a binary switch between neuronal and glial determination in Drosophila. Cell 82: 1025–1036. 7553844 10.1016/0092-8674(95)90281-3

[2] Jones BW, Fetter RD, Tear G, Goodman CS (1995) glial cells missing: a genetic switch that controls glial versus neuronal fate. Cell 82: 1013–1023. 7553843 10.1016/0092-8674(95)90280-5

[3] Vincent S, Vonesch JL, Giangrande A (1996) Glide directs glial fate commitment and cell fate switch between neurones and glia. Development 122: 131–139. 8565824 10.1242/dev.122.1.131

[4] Akiyama-Oda Y, Hosoya T, Hotta Y (1998) Alteration of cell fate by ectopic expression of Drosophila glial cells missing in non-neural cells. Dev Genes Evol 208: 578–585. 9811976 10.1007/s004270050217

[5] Bernardoni R, Miller AA, Giangrande A (1998) Glial differentiation does not require a neural ground state. Development 125: 3189–3200. 9671591 10.1242/dev.125.16.3189

[6] Flici H, Erkosar B, Komonyi O, Karatas OF, Laneve P, et al (2011) Gcm/Glide-dependent conversion into glia depends on neural stem cell age, but not on division, triggering a chromatin signature that is conserved in vertebrate glia. Development 138: 4167–4178. 21852399 10.1242/dev.070391

[7] Miller AA, Bernardoni R, Giangrande A (1998) Positive autoregulation of the glial promoting factor glide/gcm. Embo J 17: 6316–6326. 9799239 10.1093/emboj/17.21.6316 PMC1170956

[8] Ragone G, Van De Bor V, Sorrentino S, Kammerer M, Galy A, et al (2003) Transcriptional regulation of glial cell specification. Dev Biol 255: 138–150. 12618139 10.1016/s0012-1606(02)00081-7

[9] De Iaco R, Soustelle L, Kammerer M, Sorrentino S, Jacques C, et al (2006) Huckebein-mediated autoregulation of Glide/Gcm triggers glia specification. Embo J 25: 244–254. 16362045 10.1038/sj.emboj.7600907 PMC1356350

[10] Soustelle L, Roy N, Ragone G, Giangrande A (2008) Control of gcm RNA stability is necessary for proper glial cell fate acquisition. Mol Cell Neurosci 37: 657–662. 18313940 10.1016/j.mcn.2007.11.007

[11] Ho MS, Chen H, Chen M, Jacques C, Giangrande A, et al (2009) Gcm protein degradation suppresses proliferation of glial progenitors. Proc Natl Acad Sci U S A 106: 6778–6783. 19346490 10.1073/pnas.0808899106 PMC2672493

[12] Schuettengruber B, Martinez AM, Iovino N, Cavalli G (2011) Trithorax group proteins: switching genes on and keeping them active. Nat Rev Mol Cell Biol 12: 799–814. 22108599 10.1038/nrm3230

[13] Ringrose L, Paro R (2007) Polycomb/Trithorax response elements and epigenetic memory of cell identity. Development 134: 223–232. 17185323 10.1242/dev.02723

[14] Schuettengruber B, Chourrout D, Vervoort M, Leblanc B, Cavalli G (2007) Genome regulation by polycomb and trithorax proteins. Cell 128: 735–745. 17320510 10.1016/j.cell.2007.02.009

[15] Schuettengruber B, Cavalli G (2009) Recruitment of polycomb group complexes and their role in the dynamic regulation of cell fate choice. Development 136: 3531–3542. 19820181 10.1242/dev.033902

[16] Kharchenko PV, Alekseyenko AA, Schwartz YB, Minoda A, Riddle NC, et al (2010) Comprehensive analysis of the chromatin landscape in Drosophila melanogaster. Nature 471: 480–485. 21179089 10.1038/nature09725 PMC3109908

[17] Enderle D, Beisel C, Stadler MB, Gerstung M, Athri P, et al (2011) Polycomb preferentially targets stalled promoters of coding and noncoding transcripts. Genome Res 21: 216–226. 21177970 10.1101/gr.114348.110 PMC3032925

[18] Brookes E, de Santiago I, Hebenstreit D, Morris KJ, Carroll T, et al (2012) Polycomb Associates Genome-wide with a Specific RNA Polymerase II Variant, and Regulates Metabolic Genes in ESCs. Cell Stem Cell 10: 157–170. 22305566 10.1016/j.stem.2011.12.017 PMC3682187

[19] Touma JJ, Weckerle FF, Cleary MD (2012) Drosophila Polycomb complexes restrict neuroblast competence to generate motoneurons. Development 139: 657–666. 22219354 10.1242/dev.071589

[20] Van De Bor V, Heitzler P, Leger S, Plessy C, Giangrande A (2002) Precocious expression of the Glide/Gcm glial-promoting factor in Drosophila induces neurogenesis. Genetics 160: 1095–1106. 11901125 10.1093/genetics/160.3.1095 PMC1462002

[21] Dominguez M, Campuzano S (1993) asense, a member of the Drosophila achaete-scute complex, is a proneural and neural differentiation gene. Embo J 12: 2049–2060. 8491195 10.1002/j.1460-2075.1993.tb05854.x PMC413427

[22] Brand M, Jarman AP, Jan LY, Jan YN (1993) asense is a Drosophila neural precursor gene and is capable of initiating sense organ formation. Development 119: 1–17. 8565817 10.1242/dev.119.1.1

[23] Altenhein B, Becker A, Busold C, Beckmann B, Hoheisel JD, et al (2006) Expression profiling of glial genes during Drosophila embryogenesis. Dev Biol 296: 545–560. 16762338 10.1016/j.ydbio.2006.04.460

[24] Papoulas O, Beek SJ, Moseley SL, McCallum CM, Sarte M, et al (1998) The Drosophila trithorax group proteins BRM, ASH1 and ASH2 are subunits of distinct protein complexes. Development 125: 3955–3966. 9735357 10.1242/dev.125.20.3955

[25] Collins RT, Furukawa T, Tanese N, Treisman JE (1999) Osa associates with the Brahma chromatin remodeling complex and promotes the activation of some target genes. Embo J 18: 7029–7040. 10601025 10.1093/emboj/18.24.7029 PMC1171766

[26] Badenhorst P, Voas M, Rebay I, Wu C (2002) Biological functions of the ISWI chromatin remodeling complex NURF. Genes Dev 16: 3186–3198. 12502740 10.1101/gad.1032202 PMC187504

[27] Jacques C, Soustelle L, Nagy I, Diebold C, Giangrande A (2009) A novel role of the glial fate determinant glial cells missing in hematopoiesis. Int J Dev Biol 53: 1013–1022. 19598118 10.1387/ijdb.082726cj

[28] Czermin B, Melfi R, McCabe D, Seitz V, Imhof A, et al (2002) Drosophila enhancer of Zeste/ESC complexes have a histone H3 methyltransferase activity that marks chromosomal Polycomb sites. Cell 111: 185–196. 12408863 10.1016/s0092-8674(02)00975-3

[29] Mohan M, Herz HM, Smith ER, Zhang Y, Jackson J, et al The COMPASS family of H3K4 methylases in Drosophila. Mol Cell Biol 31: 4310–4318. 21875999 10.1128/MCB.06092-11 PMC3209330

[30] Grimaud C, Negre N, Cavalli G (2006) From genetics to epigenetics: the tale of Polycomb group and trithorax group genes. Chromosome Res 14: 363–375. 16821133 10.1007/s10577-006-1069-y

[31] Tie F, Banerjee R, Stratton CA, Prasad-Sinha J, Stepanik V, et al (2009) CBP-mediated acetylation of histone H3 lysine 27 antagonizes Drosophila Polycomb silencing. Development 136: 3131–3141. 19700617 10.1242/dev.037127 PMC2730368

[32] Kwong C, Adryan B, Bell I, Meadows L, Russell S, et al (2008) Stability and dynamics of polycomb target sites in Drosophila development. PLoS Genet 4: e1000178 doi:10.1371/journal.pgen.1000178. 18773083 10.1371/journal.pgen.1000178 PMC2525605

[33] Schuettengruber B, Ganapathi M, Leblanc B, Portoso M, Jaschek R, et al (2009) Functional anatomy of polycomb and trithorax chromatin landscapes in Drosophila embryos. PLoS Biol 7: e13 doi:10.1371/journal.pbio.1000013.. 19143474 10.1371/journal.pbio.1000013 PMC2621266

[34] Schwartz YB, Kahn TG, Stenberg P, Ohno K, Bourgon R, et al (2010) Alternative epigenetic chromatin states of polycomb target genes. PLoS Genet 6: e1000805 doi:10.1371/journal.pgen.1000805.. 20062800 10.1371/journal.pgen.1000805 PMC2799325

[35] Kammerer M, Giangrande A (2001) Glide2, a second glial promoting factor in Drosophila melanogaster. Embo J 20: 4664–4673. 11532931 10.1093/emboj/20.17.4664 PMC125586

[36] Gindhart JG Jr, Kaufman TC (1995) Identification of Polycomb and trithorax group responsive elements in the regulatory region of the Drosophila homeotic gene Sex combs reduced. Genetics 139: 797–814. 7713433 10.1093/genetics/139.2.797 PMC1206382

[37] Kassis JA (2002) Pairing-sensitive silencing, polycomb group response elements, and transposon homing in Drosophila. Adv Genet 46: 421–438. 11931233 10.1016/s0065-2660(02)46015-4

[38] Paladi M, Tepass U (2004) Function of Rho GTPases in embryonic blood cell migration in Drosophila. J Cell Sci 117: 6313–6326. 15561773 10.1242/jcs.01552

[39] Soustelle L, Trousse F, Jacques C, Ceron J, Cochard P, et al (2007) Neurogenic role of Gcm transcription factors is conserved in chicken spinal cord. Development 134: 625–634. 17215311 10.1242/dev.02750

[40] Soustelle L, Giangrande A (2007) Glial differentiation and the Gcm pathway. Neuron Glia Biol 3: 5–16. 18634574 10.1017/S1740925X07000464

[41] Van De Bor V, Walther R, Giangrande A (2000) Some fly sensory organs are gliogenic and require glide/gcm in a precursor that divides symmetrically and produces glial cells. Development 127: 3735–3743. 10934018 10.1242/dev.127.17.3735

[42] Bernardoni R, Vivancos V, Giangrande A (1997) glide/gcm is expressed and required in the scavenger cell lineage. Dev Biol 191: 118–130. 9356176 10.1006/dbio.1997.8702

[43] Chotard C, Leung W, Salecker I (2005) glial cells missing and gcm2 cell autonomously regulate both glial and neuronal development in the visual system of Drosophila. Neuron 48: 237–251. 16242405 10.1016/j.neuron.2005.09.019

[44] Soustelle L, Giangrande A (2007) Novel gcm-dependent lineages in the postembryonic nervous system of Drosophila melanogaster. Dev Dyn 236: 2101–2108. 17654713 10.1002/dvdy.21232

[45] Akiyama Y, Hosoya T, Poole AM, Hotta Y (1996) The gcm-motif: a novel DNA-binding motif conserved in Drosophila and mammals. Proc Natl Acad Sci U S A 93: 14912–14916. 8962155 10.1073/pnas.93.25.14912 PMC26236

[46] Andersson ER, Sandberg R, Lendahl U (2011) Notch signaling: simplicity in design, versatility in function. Development 138: 3593–3612. 21828089 10.1242/dev.063610

[47] Tamkun JW, Deuring R, Scott MP, Kissinger M, Pattatucci AM, et al (1992) brahma: a regulator of Drosophila homeotic genes structurally related to the yeast transcriptional activator SNF2/SWI2. Cell 68: 561–572. 1346755 10.1016/0092-8674(92)90191-e

[48] Collins RT, Treisman JE (2000) Osa-containing Brahma chromatin remodeling complexes are required for the repression of wingless target genes. Genes Dev 14: 3140–3152. 11124806 10.1101/gad.854300 PMC317146

[49] Marenda DR, Zraly CB, Dingwall AK (2004) The Drosophila Brahma (SWI/SNF) chromatin remodeling complex exhibits cell-type specific activation and repression functions. Dev Biol 267: 279–293. 15013794 10.1016/j.ydbio.2003.10.040

[50] Breiling A, O'Neill LP, D'Eliseo D, Turner BM, Orlando V (2004) Epigenome changes in active and inactive polycomb-group-controlled regions. EMBO Rep 5: 976–982. 15448640 10.1038/sj.embor.7400260 PMC1299157

[51] Papp B, Muller J (2006) Histone trimethylation and the maintenance of transcriptional ON and OFF states by trxG and PcG proteins. Genes Dev 20: 2041–2054. 16882982 10.1101/gad.388706 PMC1536056

[52] Dejardin J, Rappailles A, Cuvier O, Grimaud C, Decoville M, et al (2005) Recruitment of Drosophila Polycomb group proteins to chromatin by DSP1. Nature 434: 533–538. 15791260 10.1038/nature03386

[53] Cabrera CV, Botas J, Garcia-Bellido A (1985) Distribution of Ultrabithorax proteins in mutants of Drosophila bithorax complex and its transregulatory genes. Nature 318: 569.

[54] Busturia A, Lloyd A, Bejarano F, Zavortink M, Xin H, et al (2001) The MCP silencer of the Drosophila Abd-B gene requires both Pleiohomeotic and GAGA factor for the maintenance of repression. Development 128: 2163–2173. 11493537 10.1242/dev.128.11.2163

[55] Bracken AP, Helin K (2009) Polycomb group proteins: navigators of lineage pathways led astray in cancer. Nat Rev Cancer 9: 773–784. 19851313 10.1038/nrc2736

[56] Pereira JD, Sansom SN, Smith J, Dobenecker MW, Tarakhovsky A, et al (2010) Ezh2, the histone methyltransferase of PRC2, regulates the balance between self-renewal and differentiation in the cerebral cortex. Proc Natl Acad Sci U S A 107: 15957–15962. 20798045 10.1073/pnas.1002530107 PMC2936600

[57] Hirabayashi Y, Suzki N, Tsuboi M, Endo TA, Toyoda T, et al (2009) Polycomb limits the neurogenic competence of neural precursor cells to promote astrogenic fate transition. Neuron 63: 600–613. 19755104 10.1016/j.neuron.2009.08.021

[58] Liu J, Casaccia P (2010) Epigenetic regulation of oligodendrocyte identity. Trends Neurosci 33: 193–201. 20227775 10.1016/j.tins.2010.01.007 PMC2849857

[59] Lavrov S, Dejardin J, Cavalli G (2004) Combined immunostaining and FISH analysis of polytene chromosomes. Methods Mol Biol 247: 289–303. 14707354 10.1385/1-59259-665-7:289

[60] Schneider I (1972) Cell lines derived from late embryonic stages of Drosophila melanogaster. J Embryol Exp Morphol 27: 353–365. 4625067

