# Supplementary figures and images for: Polycomb Controls Gliogenesis by Regulating the Transient Expression of the Gcm/Glide Fate Determinant
Source: PLoS Genet. 2012 Dec 27;8(12):e1003159. doi: 10.1371/journal.pgen.1003159 (PMC3531469; doi:10.1371/journal.pgen.1003159)

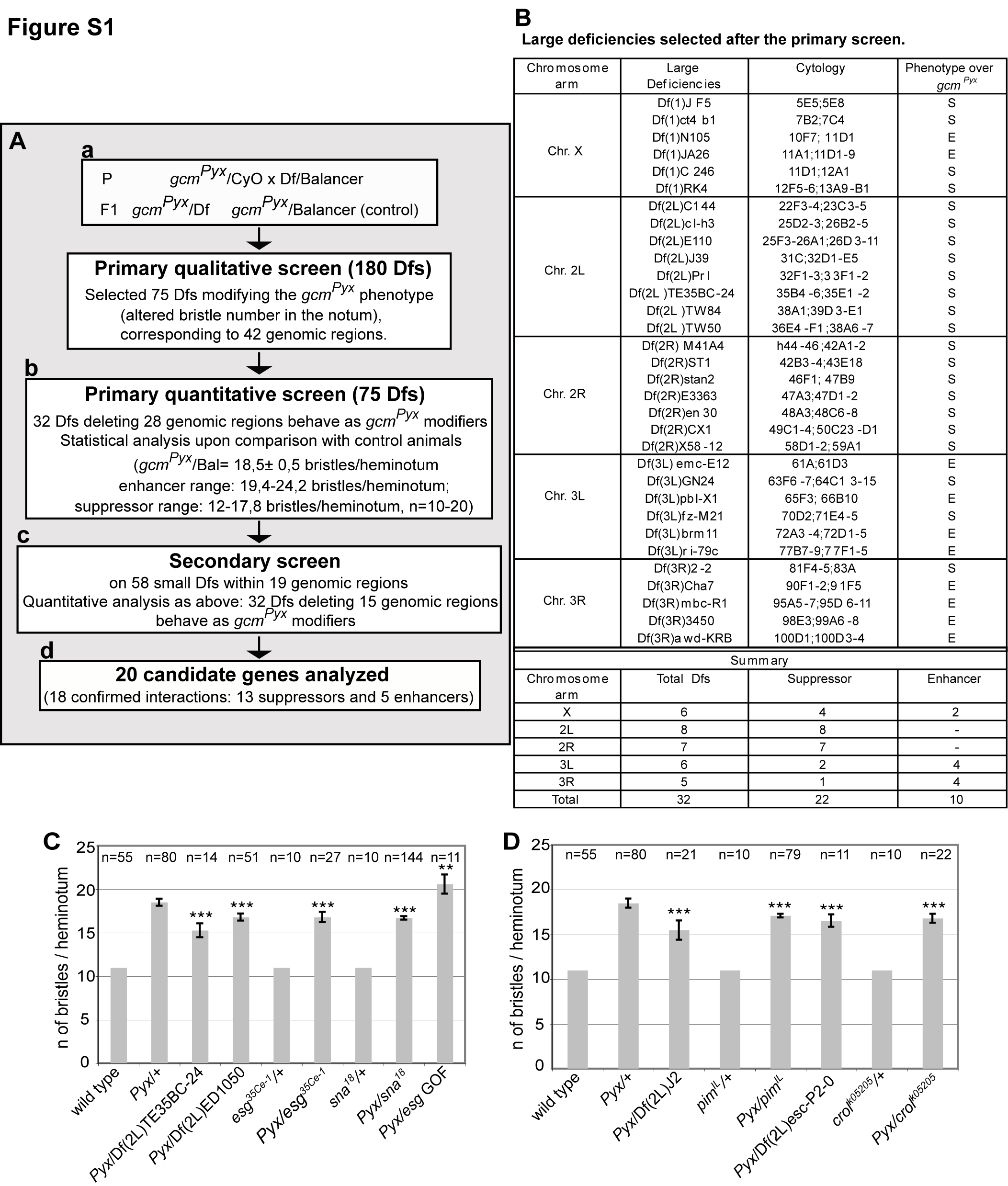

Supplement: Figure S1 — Genetic screen for gcmPyx modifiers and validation of candidate genes. (A) Flow-chart of the screen: gcmPyx/CyO, twist-LacZ flies were crossed to Bloomington Deficiency kit strains. The bristle phenotype was compared between sibs: control (gcmPyx/Balancer) and experimental females (gcmPyx/Df). The screen was performed in three steps (primary qualitative, primary quantitative, secondary) and followed by gene validation. The number of analyzed deficiencies and the quantitative data are presented. Bal = balancer, Dfs = Deficiencies. (B) Primary quantitative screen deficiencies summary. Top: chromosome arms, names and cytology of deficiencies selected as strong modifiers of the bristle phenotype: suppressors (S) and enhancers (E). Bottom: total number of modifier deficiencies on each chromosome arm, number of suppressor and enhancer deficiencies. (C) Histograms present the average number of bristles per heminotum (y-axis) in different genotypes (x-axis). Large (Df(2L)TE35BC-24) and small Df(2L)ED1050) deficiencies cover esg, wor and sna genes. Pyx/esg GOF stands for gcmPyx/EP(2)0684; hs-Gal4. Phenotype observed upon heat shocking gcmPyx animals that carry the hs-Gal4 driver and the EP(2)0684 insertion expressing esg in response to Gal4 induction. Note that both deficiencies eliminate wor, esg and sna, but only the large one covers the Su(H) mutation, which acts as suppressor. This may explain why the large deficiency seems to act as a stronger suppressor. (C) Deficiencies deleting pim and crol genes as well as their single mutations. In each graph, average values are indicated +/− SEM (bars); P-values from t-test are indicated in the following way: *** (P≤10−3), ** (P≤10−2), * (P≤5×10^−2^). P-values vs. gcmPyx/+: gcmPyx/Df(2L)TE35BC-24 (9,3×10^−7^); gcmPyx/Df(2L)ED1050 (4,7×10^−8^); gcmPyx/esg (5,4×10^−6^); gcmPyx/sna (1,3×10^−18^); Pyx/esg GOF (0,005). gcmPyx/Df(2L)J2 (4,7×10^−8^); gcmPyx/pim (5×10^−5^); gcmPyx/Df(2L)esc-P2-0 (6,8×10^−5^); gcmPyx/crol (8,3×10^−7^). (TIF) [file pgen.1003159.s001.tif]

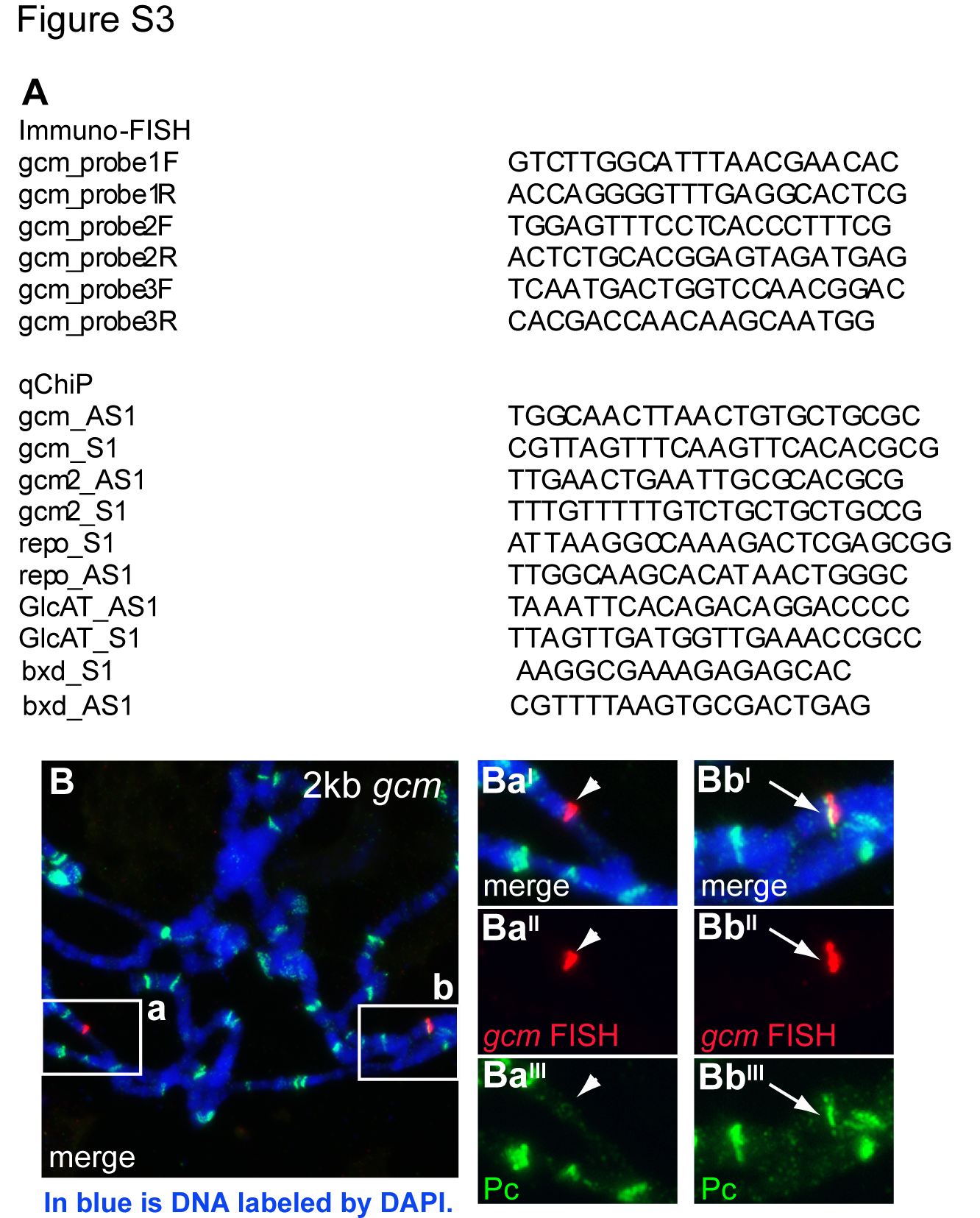

Supplement: Figure S3 — The Pc binding region and the polytene chromosomes of the 2 kb transgenic line. (A) List of primers used for immuno-FISH and qCHIP. (B) Immuno-FISH staining (anti-Pc, gcm) on polytene chromosomes carrying a transgene including a 2 kb region upstream of the gcm transcription start site. (TIF) [file pgen.1003159.s003.tif]

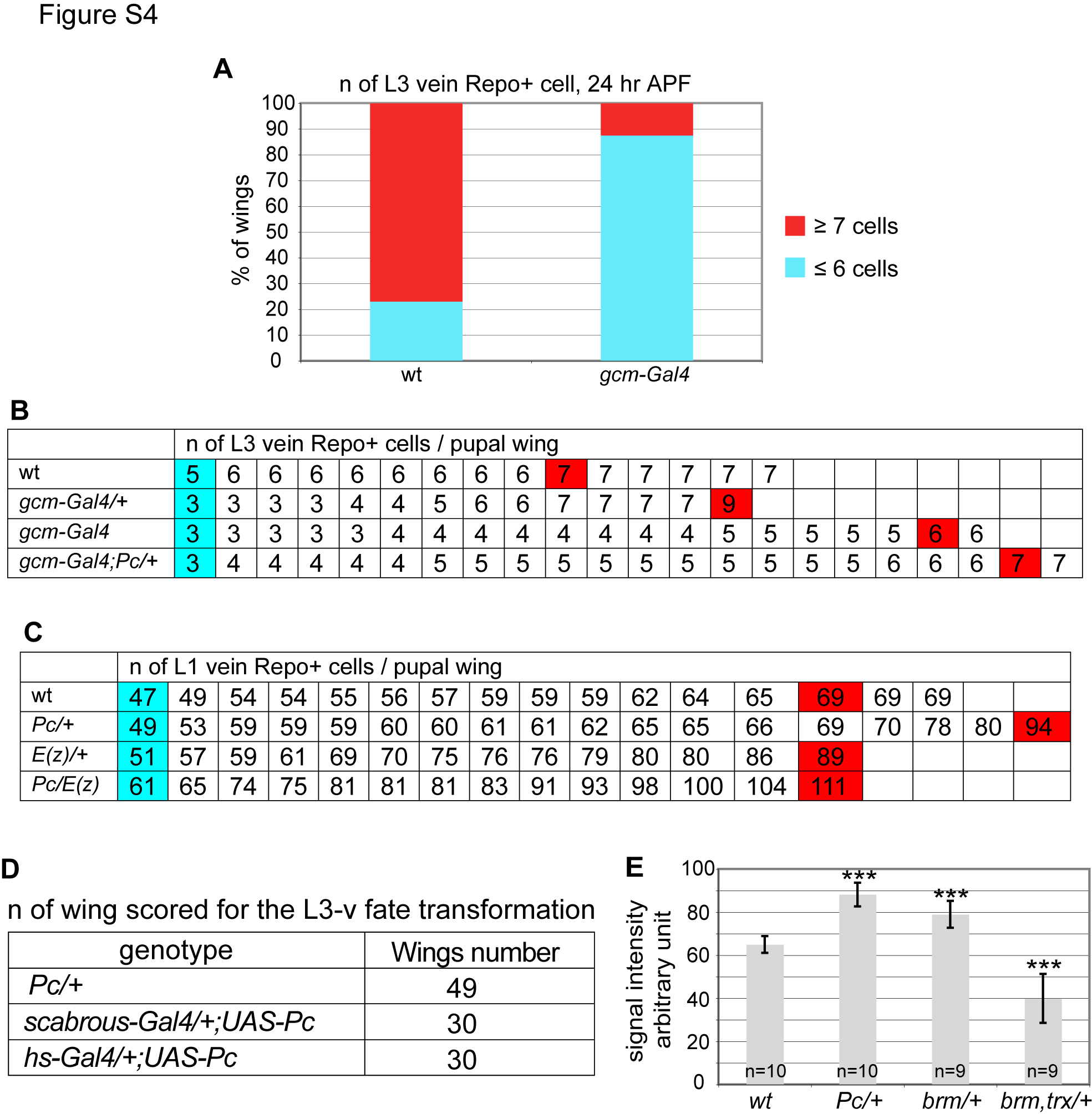

Supplement: Figure S4 — Mutant phenotypes in wings and brains. (A) Percentage of 24 hr APF wings of the described genotypes carrying different numbers of Repo+ cells on the L3 vein. (B,C) Summary tables showing the number of Repo+ cells observed on the L3 (B) and L1 veins (C) in each pupal wing analyzed. The observed minimum and maximum value in samples of the different genotypes are respectively highlighted in pale-blue and red. (D) Number of wings scored for the fate transformation phenotype in heterozygous Pc/+ wings or in Pc overexpressing wings, using two different Gal4 drivers. (E) Quantitative analysis of gcm expression in the optic lobe (see Figure 5J–5H): histograms present the average signal intensity (y-axis) in the different genotypes (x-axis). P values of t-test vs. wt: Pc/+ (1,7×10^−6^); brm/+(0,0009); brm,trx/+(0,0008). (TIF) [file pgen.1003159.s004.tif]

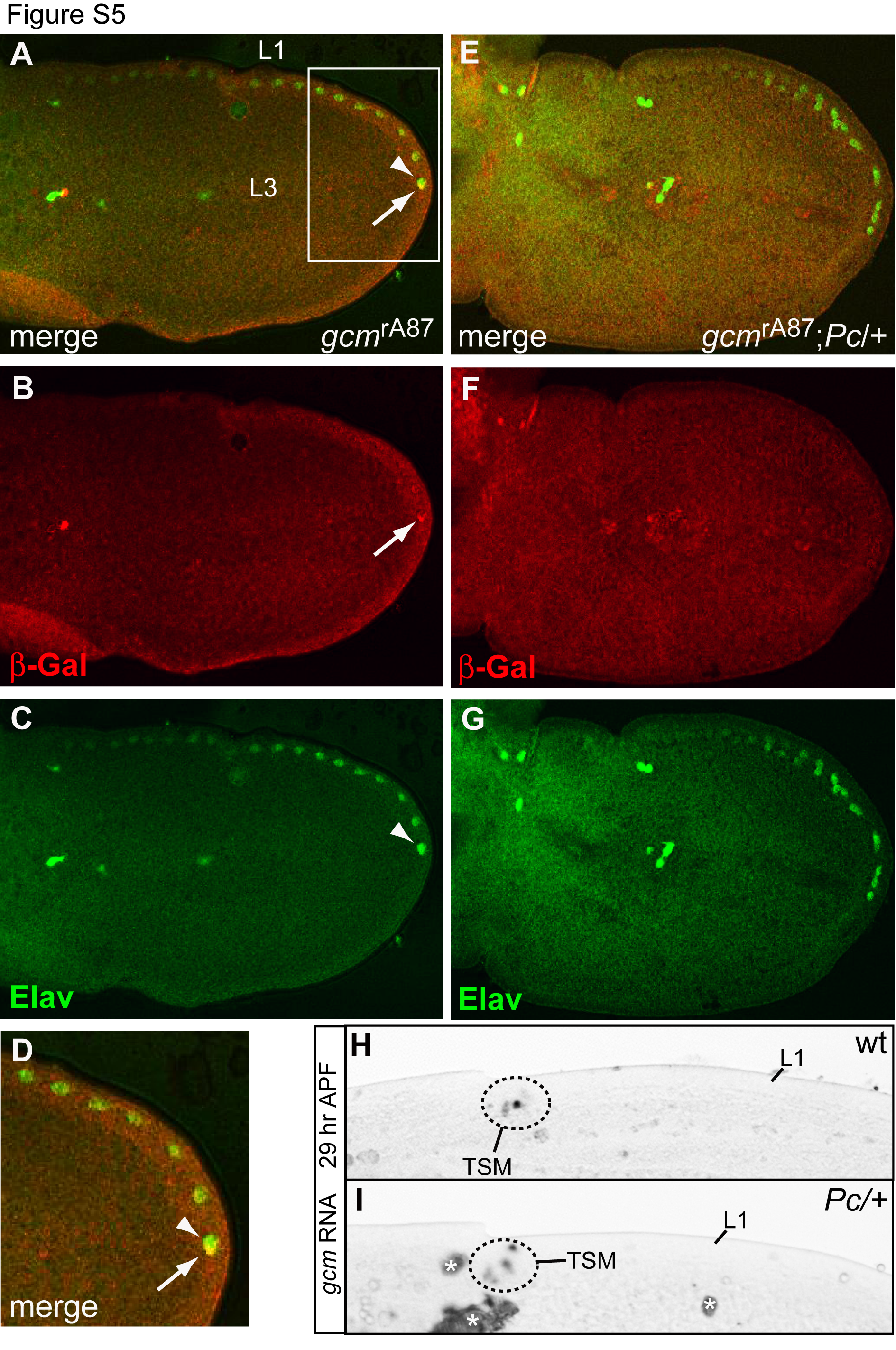

Supplement: Figure S5 — Initiation and extinction of gcm expression in Pc/+ wings. (A–G) Immunolabeling of 7 hr APF wings from the P-mediated insertional gcmrA87 allele expressing the LacZ reporter, anterior to the top, distal to the right. By this stage, the β-Gal labeling is still not present onto the L1 vein in most of the wings (11/12); in one wing (A–D), one β-Gal labeled cell is visible at the distal tip (β-Gal in red, neuronal labeling (Elav) in green). This cell (arrow) is close to a neuron (arrowhead), (D) shows a magnification from the boxed region. L1 and L3 indicate the position of the L1 and L3 veins, respectively. In wings heterozygous for Pc (n = 13) (E–G), no precocious β-Gal labeling was observed on the L1 vein. (H,I) In situ hybridization with a gcm-specific probe on 29 h APF wings from wt (H) and from Pc/+ (I) animals. Note that, in both backgrounds, gcm is no more expressed. (TIF) [file pgen.1003159.s005.tif]

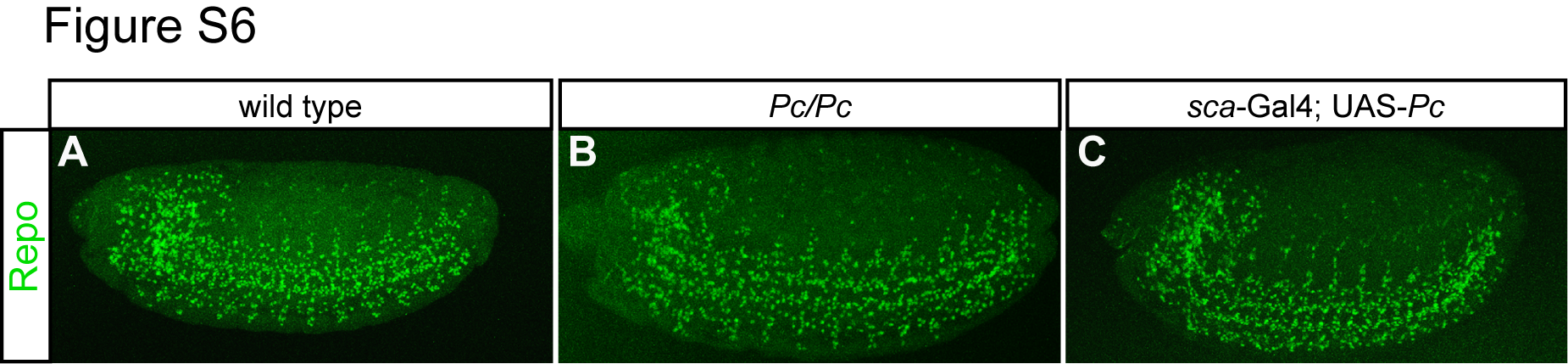

Supplement: Figure S6 — Repo expression in wild type, Pc LOF and GOF. Immunolabeling to show Repo protein in st. 14 embryos. Ventrolateral view in wild type (A), Pc/Pc (B), scabrous-Gal4/UAS-Pc animals (C). (TIF) [file pgen.1003159.s006.tif]

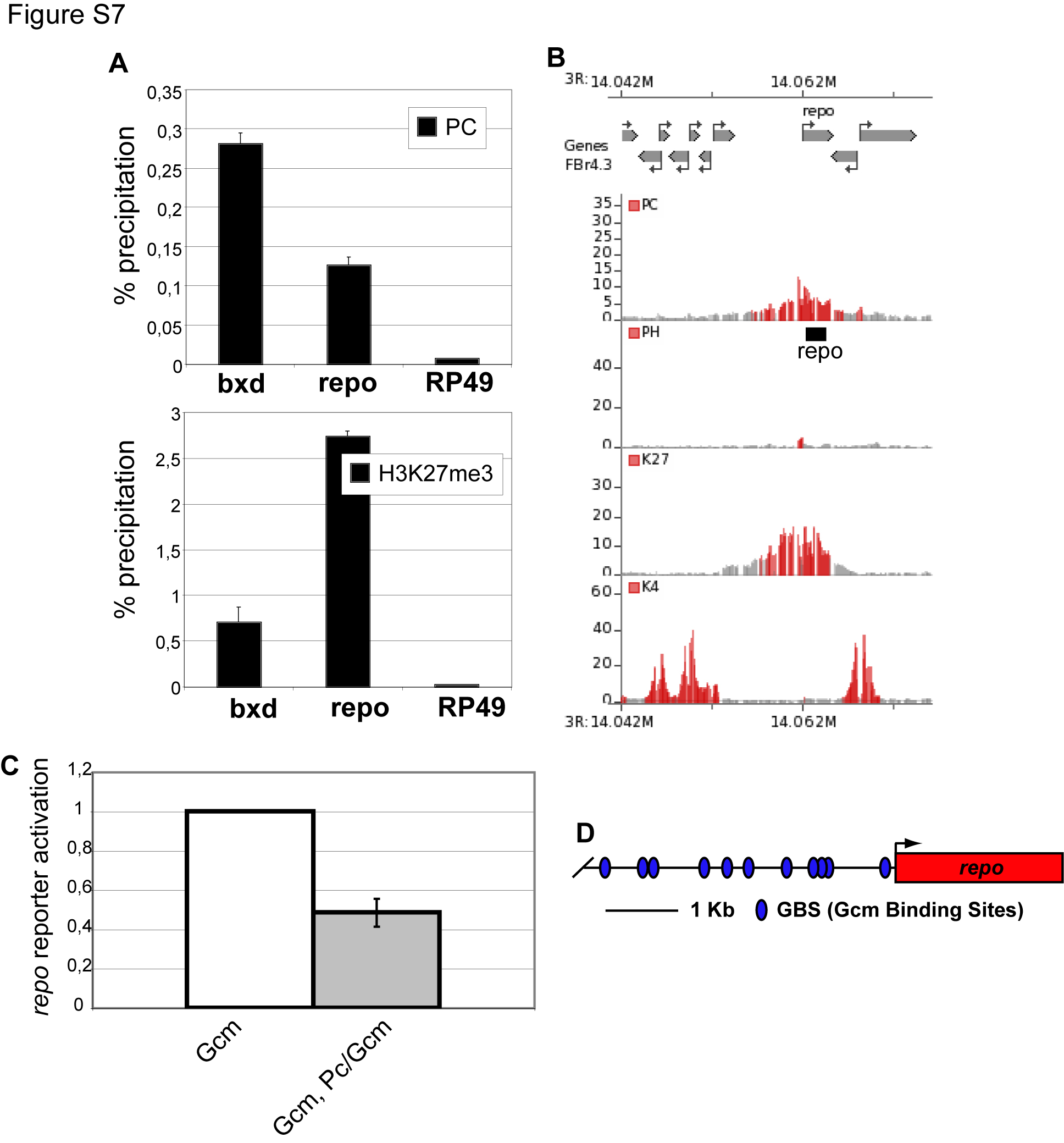

Supplement: Figure S7 — Pc binds to and acts on the repo promoter. (A) Levels of Pc binding and H3K27me3 at the repo locus in Drosophila embryos were determined by quantitative ChIP (qChIP) experiments, the bxd locus was used as a positive control. Results are represented as percentage of input chromatin precipitated. The standard deviation was calculated from two independent experiments. (B) ChIP-on-chip binding profiles of indicated PcG proteins and histone marks in Drosophila embryos at the repo regulatory region obtained as reported by [33]. The plots show the ratios (fold change) of specific IP versus mock IP assays. Significantly enriched fragments (P-value<1×10^−4^) are shown in red. Black bars indicate the location of primers used for qChIP analysis. The graph (C) shows the activation of a reporter construct carrying 4 kb from the repo upstream regulatory sequence displaying eleven GBSs (D). The ratio between reporter activity upon Gcm/Pc coexpression and that observed when only Gcm is expressed indicates that the repo promoter is activated when Gcm is expressed in S2 cells and repressed upon Gcm and Pc coexpression. (TIF) [file pgen.1003159.s007.tif]

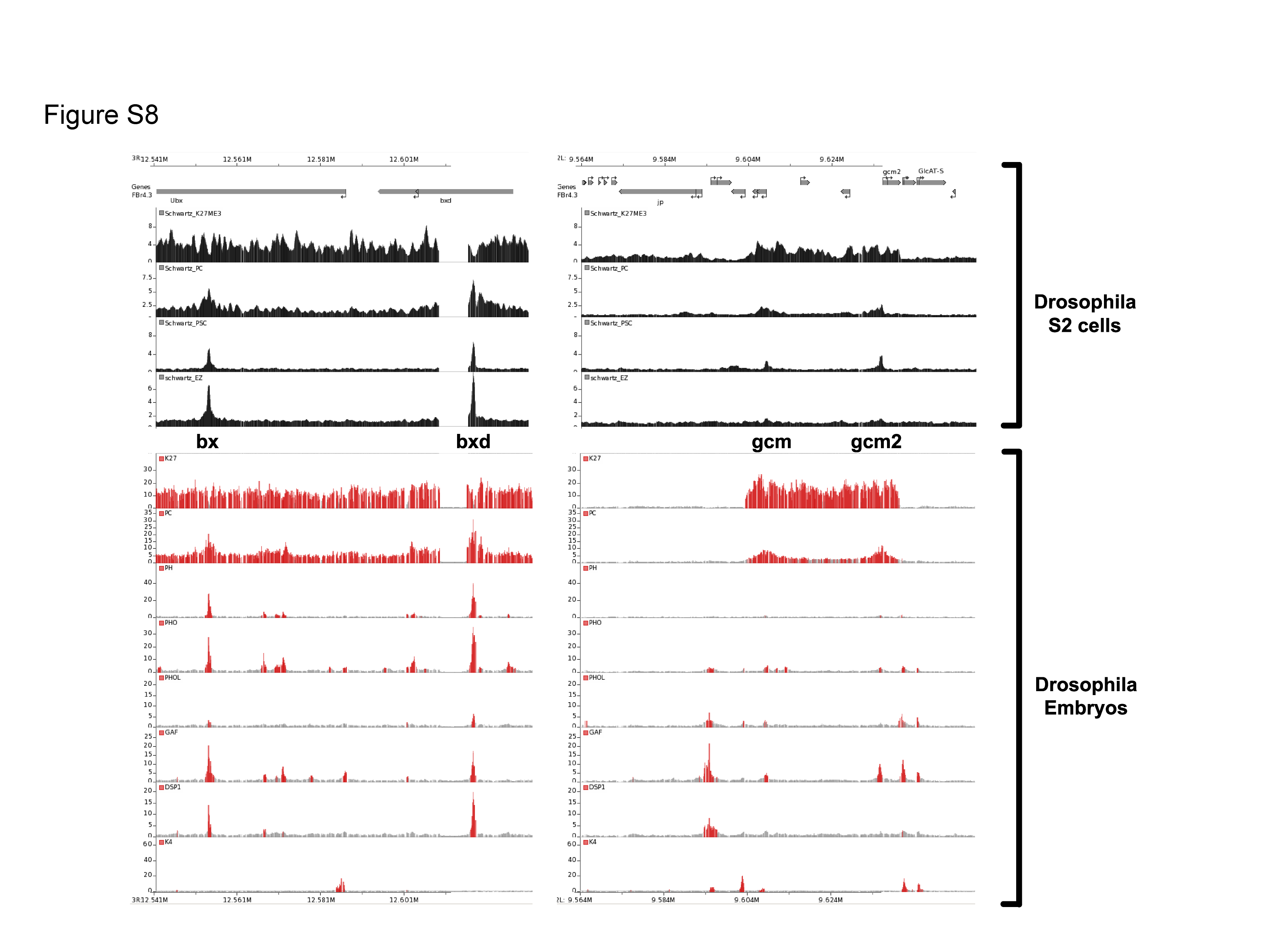

Supplement: Figure S8 — Comparison between gcm/gcm2 and bx/bxd PREs. ChIP-on-chip binding profiles of indicated PcG proteins and histone marks in Drosophila S2 cells from (Schwartz et al., 2006) or Drosophila embryos from (Schuettengruber et al., 2009). Nomenclature as in Figure 2. (TIF) [file pgen.1003159.s008.tif]
