# Supplementary material for: Polycomb Controls Gliogenesis by Regulating the Transient Expression of the Gcm/Glide Fate Determinant
Source: PLoS Genet. 2012 Dec 27;8(12):e1003159. doi: 10.1371/journal.pgen.1003159 (PMC3531469; doi:10.1371/journal.pgen.1003159)
Supplement: Figure S2 — Summary of the genes analyzed over gcmPyx. From left to right, columns indicate the name of the gene, the cytology, the heterozygous phenotype over gcmPyx (No – no effect; S – suppressor; E – enhancer), the phenotype of the large deficiency over gcmPyx (nt – the gene region is not covered by the tested deficiencies), the function of the gene, previous identification as a Gcm target/regulator, references. TF = transcription factor, TrxG = Trithorax group, PcG = Polycomb group; JAK-STAT = Janus kinase/Signal Transducer and Activator of Transcription. (PDF) [file pgen.1003159.s002.pdf]

Figure S2 Summary of the genes analyzed over *gcm*^{Pyx}.

Gene name	Cytology	Mutant Effect	Large Df Effect	Function	Microarray DAM- ID	Reference
**Genes playing a role in the nervous system**						
midline (mid)	25E2	S	S (Table 1)	TF; segment polarity gene; neuroblast specification		Buescher et al. 2006
escargot (esg)	35D2	S	S (Table 1)	TF; neuroblast asymmetric division		Cai et al. 2003
worniu (wor)	35D2	No	S (Table 1)	TF; neuroblast asymmetric division		Cai et al. 2003
snail (sna)	35D2	S	S (Table 1)	TF; neuroblast asymmetric division	*gcm* is a Snail target (DAM-ID)	Cai et al. 2003
brain tumor (brat)	37C1-C6	S	S (Table 1)	Translational repressor; proliferation control of specific larval neuroblasts		Bestschinger et al. 2006; Bello et al. 2006
huckebein (hkb)	82A4	Weak S	S (Table 1)	TF; positive cofactor of *gcm* autoregulation		De Iaco et al. 2006
prospero (pros)	86E2-86E4	Weak S	Weak E	TF; choice between stem cell renewal and differentiation	*gcm* is a Prospero target (DAM-ID); *pros* is dowregulated in *gcm LOF* embryos	Choksi et al. 2006 Ragone et al., 2001
fruitless (fru)	91A7-91B3	Weak E	E (Table 1)	TF; axonal tracks formation		Song et al. 2002
**Chromatin structure**						
Mi2 (Mi2)	76D3-D4	E	nt	TrxG protein (CHD protein, NuRD complex), chromatin remodeling		Reviewed in Clapier and Cairns 2009; Shuettengruber et al.

						2011
Enhancer of bithorax (E(bx)) (NURF301)	61B3	E	E (Table 1)	TrxG protein (ISWI complex); chromatin remodeling; involved in the JAK-STAT hematopoietic pathway		Badenhorst et al. 2002
brahma (brm)	72C1	E	E (Table 1)	TrxG protein (SWI/SNF complex); chromatin remodeling	Brm associated protein Bap60 is upregulated upon *gcm* overexpression (Egger et al. 2002)	Reviewed in Grimaud et al. 2006 and Shuettengruber et al. 2011
osa (osa)	90C1-C2	E	nt	TrxG protein (SWI/SNF complex); Brm partner		Reviewed in Grimaud et al. 2006 and Shuettengruber et al. 2011
absent, small, or homeotic discs 1(ash1)	76B8-B9	S	nt	TrxG protein (Ash1 complex); histone methyltransferase activity		Reviewed in Grimaud et al. 2006 and Shuettengruber et al. 2011
nejire (nej)(dCBP)	8F7-F9	S	No	TrxG protein (TAC1 and Ash1 complexes); H3K27 acetylation		Reviewed in Grimaud et al. 2006 and Shuettengruber et al. 2011
trithorax (Trx)	88B1	S	No	TrxG protein (TAC1); H3K4 methylation; transcription activator		Reviewed in Grimaud et al. 2006 and Shuettengruber et al. 2011
Polycomb (Pc)	78C6	E	Weak E	PcG protein (PRC1); recognition of H3K27 methylation mark; transcription repressor		Reviewed in Grimaud et al. 2006
Enhancer of zeste (E(z))	67E5	E	No	PcG protein (PRC2); H3K27 methylation		Reviewed in Grimaud et al. 2006

extra sexcombs (esc)	33A2	E	S	PcG protein (PRC2)		Reviewed in Grimaud et al. 2006
pipsqueak (psq)	47A13-B1	E	S	PcG recruiter		Reviewed in Grimaud et al. 2006
lilliputian (lilli)	23C1-C3	S	S (Table 1)	High Mobility Group-protein		VanderZwan-Butler et al. 2007
**Notch pathway**						
Suppressor of Hairless (Su(H))	35B8	S	S (Table 1)	Transcriptional repression/activation of Notch-target genes		Castro et al. 2005
groucho (gro)	96F10	S	nt	Transcriptional repression of Notch-target genes		Castro et al. 2005
***Notch* (N)**	3C7-C9	E	nt	Transmembrane receptor; transcriptional activation		Reviewed in Artavanis-Tsakonas et al. 1999; Tien et al. 2009
Delta (Dl)	92A1-A2	E	No	Notch-ligand		Reviewed in Le Borgne 2005
lethal (2) giant discs (l(2)gd1)	32D3-D4	S	S (Table 1)	Notch receptor trafficking; negative regulation of Notch pathway		Childresse et al 2006
Enhancer of split (E(spl))	96F10	E	nt	TF; transcription repressor		Kageyama et al. 2008
cut (ct)	7B4-7B6	S	S (small Df not tested)	TF; negative regulation of Notch pathway		Sun and Deng 2005
**Gcm known targets**						
reverse polarity (repo)	90F9-F10	Weak E	E (Table 1)	TF; glial differentiation	Upregulated by Gcm overexpression in microarray (Egger et al. 2002; Freeman et al. 2003)	Halter et al. 1995

tramtrack (ttk)	100D1	E	E (Table 1)	TF; neuronal repressor		Giesen et al. 1996, Badenhorst 2001
pimples (pim)	31D10	S	S (Table 1)	sister chromatid segregation	Upregulated by *gcm* overexpression in microarray (Altenhein et al. 2006)	Stratmann and Lehner 1996
crooked legs (crol)	33A1-A2	S	S (Table 1)	TF; PNS development	Downregulated by *gcm* loss-of-function in microarray (Altenhein et al. 2006)	Norga et al. 2003
E2F transcription factor 2 (E2f2)	39A5-A6	S	S (Table 1)	TF; transcription repressor	Downregulated by Gcm overexpression in microarray (Egger et al, 2002)	Frolov et al. 2001
longitudinals lacking (lola)	47A11-A13	S	S (Table 1)	TF; transcription repressor, axon guidance in embryonic NS	Downregulated by Gcm overexpression in microarray (Egger et al, 2002)	Ginger et al. 1996
**Small RNA metabolism**						
Argonaute 1 (Ago1)	50C9-C17	S	S (Table 1)	gene silencing by miRNA		Reviewed in Wu and Belasco 2008; Liu et al. 2008; Hock and Meister 2008
