# Supplementary material for: Polycomb Controls Gliogenesis by Regulating the Transient Expression of the Gcm/Glide Fate Determinant
Source: PLoS Genet. 2012 Dec 27;8(12):e1003159. doi: 10.1371/journal.pgen.1003159 (PMC3531469; doi:10.1371/journal.pgen.1003159)
Supplement: Text S1 — Supplementary materials and methods. (DOC) [file pgen.1003159.s009.doc]

**Supplementary material and methods**

***Drosophila* stocks and crosses**

We used the deficiency kits to screen the 1st, 2nd, 3rd and 4th chromosomes (respectively DK1, DK2, DK3 and DK4). These kits uncover 67-75% of euchromatin (<http://flystocks.bio.indiana.edu/Browse/df-dp/dfkit-history.htm>). We used 180 large deficiencies: 37 to analyze the 1st chromosome, 67 for the 2nd chromosome, 73 for the 3rd chromosome and 3 for the 4th chromosome. For the secondary deficiency screen (see Figure 1) we used deficiencies available from Umea stock center as well as DrosDel and Exelixis deficiencies obtained from Bloomington Drosophila Stock Center (listed in Table 1).

Mutant stocks used for validation of candidate genes on the 2nd or 3rd chromosome are the following: *y^1^ w^67c23^; P{w^+mC^=lacW}UbcD^2k13206^/CyO*; *mid^1^ cn^1^ bw^1^ sp^1^/CyO*; *lilli^A17-2^ cn^1^ bw^1^/CyO*; *pim^IL^ cn^1^ bw^1^ sp^1^/CyO*; *y^1^ w^67c23^; y^1^ w^67c23^; P{w^+mC^ y^+mDint2^=EPgy2}l(2)gd1^EY04750^/CyO; P{lacW}crol^k05205^/CyO; b^1^ esg^35Ce-1^ l(2)CA61^VS^ pr^1^ cn^1^ bw^1^/SM5*; *Adh^n7^ wor^1^ cn^1^ vg^1^/CyO*; *sna^18^ cn^1^ bw^1^ sp^1^/CyO; Su(H)1/In(2L)Cy, In(2R)Cy, Cy^1^ pr^1^; N^55e11^ P{ry^+t7.2^=neoFRT}19A/FM7c; Dl^3^/In(3R)C, e^1^; In(3R)E(spl)^rv1^, E(spl)^rv1^/TM6B, Tb^1^; brat^1^ rdo^1^ hk^1^ pr^1^/CyO; w^*^; E2f2^76Q1^ cn^1^ bw^1^/CyO, P{ftz/lacB}E3; cn^1^ P{PZ}lola^00642^/CyO; ry^506^; y^1^ w^67c23^; P{w^+mC^=lacW}Ago1^k08121^/CyO; brm^2^ e^s^ ca^1^/TM6B, Sb^1^ Tb^1^ ca^1^; osa^2^/TM6B, Tb^1^ ; w*; P{w^+mC^=UAS-osa}s2/CyO; w^1118^; Pc^1^/TM1; In(3R)P(Pc^3^), Pc^3^/TM1*; *w^*^; P{hsp70-CD2.J}76 kni^ri-1^ Pc^15^ P{FRT(w^hs^)}2A/TM6B, Tb^1^* ; *psq^E39^/CyO; ry^506^* ; *P{ry11}E(bx)^ry122^, mwh^1^ ry^506^ e^1^/TM3, ry^RK^ Sb^1^ Ser^1^; P{neoFRT}82B trx^E2^/TM6C, Sb^1^ Tb^1^; brm^2^ trx^E2^ ca^1^/TM6B, Tb^1^ ca^1^; w*; ash1^22^ P{w^+mW.hs^=FRT(w^hs^)}2A/TM6C, Sb^1^ Tb^1^; hkb^2^, st^1^ pp^1^, ca^1^/TM3, Sb^1^ Ser^1^; pros^17^/TM3, Ser^1^; Mi-2^4^ red^1^ e^4^/TM6B, Sb^1^ Tb^1^ ca^1^; y^1^ w^1^ ct^C145^/FM3/Dp(1;Y)ct^+^y^+^;* *w*;E(z)^731^P{1xFRT.G}2A/TM6C, Sb^1^ Tb^1^; ry^506^ P{ry^+t7.2^=PZ}fru^3^/MKRS; In(2L)t, esc^1^ c^1^ sp^1^/SM5; nej^3^/FM7; repo3^-692^ / TM3, hb-lacZ; w*; ttk^1e11^/TM3, ry* Sb^1^.*

The *hs-Gal4* strain was obtained from Bloomington Drosophila Stock Center.

*EP(2)0684* was obtained from Szeged stock center. *gro[BX22]* was from Dr Paroush, *repo3^-692^ / TM3, hb-lacZ* was from Dr Technau.

We homogenized the genetic background: firstly, we crossed males carrying a mutation with females of the double balanced stock *w^1118^; CyO/Sp; TM6, Ubx/Sb*. F1 males from this cross carrying *w^1118^, CyO* or *Sp, Sb* and a candidate gene mutation on the 2nd or 3rd chromosome were crossed with *gcm^Pyx^*/*CyO, twist-LacZ* or *gcm^Pyx^/Gla, Bc* females.

The strain containing 2 kb- and 9 kb- upstream *gcm* sequences were described in [8]. *gcm^Pyx^* is described in [20]. For *gcm* mutant *gcm-Gal4* see [38]; [44]; [39], for *gcm^rA87^* allele see [3]. The chromosome containing one dose of UAS*-gcm* was obtained by recombination from a double insert (M24A: [5]). To overexpress *gcm*, we used a *scabrous-GAL4* (*sca-GAL4*) driver which mimics the *scabrous* profile of expression in the whole neurogenic region.

The *UAS-Pc* construct was obtained by cloning the Pc cDNA into the pUAS cloning vector and injected into the germline.

**Immunolabeling and *in situ* hybridization**

The following primary antibodies were used: sheep-anti-digoxigenin (Boheringer) (1:500), mouse-anti-repo (DSHB, 8D12) (1:100), rat-anti-elav (DHSB, 7E8A10) (1:300), rabbit-anti-ß-gal (Cappel, #55976) (1:500), chicken-anti-GFP (Abcam, ab13970) (1:1000), rabbit-anti-phosphohistone H3 (Upstate biotechnology #06-570) (1:5000).

The following secondary antibodies were used at 1:600: donkey-anti-sheep IgG Cy3 (Jackson, #713-165-003), donkey-anti-mouse IgG Cy3 (Jackson, #715-165-151), donkey-anti-rabbit IgG Cy3 (Jackson, #711-165-152), goat-anti-mouse IgG Cy5 (Jackson, #115-175-100), goat-anti-rat Ig Cy5 (Jackson, #112-175-167), donkey-anti-chicken IgG FITC (Jackson, #703-095-155).

***In situ* quantification**

In situ hybridization in the larval CNS was performed as in [39]. Two parameters were used to compare *gcm* expression in different genotypes: the size of the signal area and the intensity of the signal. Five CNSs were analyzed for each genotype and the two hemispheres were analyzed separately. Preparations were scanned on a Leica SP1 confocal microscope, the Z axis was defined to include all the *gcm* specific RNA signal in the lamina region (described in [39]; [44]). The scanning step was 2,5  μm. The acquisitions were analyzed using the ImageJ software. To measure the size of the signal area, for each section, a mask was drawn around the region of the signal. Then, three sections with the largest area value were chosen, the average of the signal area was calculated and normalized by the hemisphere area. To measure the signal intensity, a rectangle mask of fixed size was used and the intensity was measured for each confocal section. Three sections with the strongest intensity were chosen to calculate the average.
